# *COX6B2* drives metabolic reprogramming toward oxidative phosphorylation to promote metastasis in pancreatic ductal cancer cells

**DOI:** 10.1038/s41389-020-0231-2

**Published:** 2020-05-15

**Authors:** Ke Nie, Jin Li, Xujun He, Yuqing Wang, Qiongya Zhao, Miaomiao Du, Hongwei Sun, Jinjing Wang, Jianxin Lyu, Hezhi Fang, Liqin Jin

**Affiliations:** 1 Key Laboratory of Laboratory Medicine, Ministry of Education, Zhejiang Provincial Key Laboratory of Medical Genetics, College of Laboratory Medicine and Life sciences, Wenzhou Medical University, Wenzhou, Zhejiang 325035 China; 2 Department of Laboratory Medicine, People’s Hospital of Changshou, Chongqing, 401220 China; 3 Zhengjiang Provincial People’s Hospital, Affiliated People’s Hospital of Hangzhou Medical College, Wenzhou, Zhejiang 310014 China; 4 The First Affiliated Hospital of Wenzhou Medical University, Wenzhou, Zhejiang 325015 China

**Keywords:** Cell migration, Cancer metabolism

## Abstract

Pancreatic ductal adenocarcinoma (PDAC) is an aggressive cancer type with poor prognosis due to its high metastatic potential, however, the role of metabolic reprogramming in the metastasis of PDAC cell is not known. Here, we report that COX6B2 drive metastasis but not cancer cell proliferation in PDAC by enhancing oxidative phosphorylation function (OXPHOS). Transcriptome and clinical analyses revealed that cytochrome c oxidase subunit 6B2 (COX6B2) positively associated with metastasis of PDAC cells. Knockdown of *COX6B2* in PDAC cells tuned down the assembly of complex IV and downregulated the function of OXPHOS, whereas re-expression of *COX6B2* restored the function of OXPHOS and metastatic potential. Mechanistically, *COX6B2* upregulated OXPHOS function to active purinergic receptor pathway for the metastasis of PDAC cells. Notably, the metastatic potential in PDAC could be reversely regulated by metformin, a drug was found accelerating the degradation of *COX6B2* mRNA in this study. Collectively, our findings indicated that a complex metabolic control mechanism might be involved in achieving the balance of metabolic requirements for both growth and metastasis in PDAC, and regulation of the expression of COX6B2 could potentially encompass one of the targets.

## Introduction

Cancer cells exhibit an altered metabolic profile when compared with normal cells^1^. In 1956, Otto Warburg first described aerobic glycolysis as this metabolic hallmark of cancer and claimed that mitochondria respiration was damaged in cancer cells^2,3^. The Warburg theory on aerobic glycolysis in cancer was advanced during the past decades: an increase in glycolysis flux facilitates glucose utilization in the pentose phosphate pathway (PPP)^4^, a metabolic pathway, which is essential for nucleotide and lipid synthesis during cell replication and provides the majority of cellular NADPH protecting cancer cells from high oxidative stress^4,5^. Accordingly, lactate, a terminal metabolite of glycolysis, was shown to promote cancer cell growth through its positive feedback role in aerobic glycolysis^6^. To date, multiple studies have agreed with the Warburg theory that mitochondria respiration becomes impaired during tumorigenesis and suggested that this impairment might promote tumor growth via changing mitochondrial to nuclear signaling pathways^7,8^; however, it was also shown that maintained mitochondrial respiration was essential for tumorigenesis^9^. This finding was attributed to the possibility that mitochondrial respiration might support the biosynthesis of metabolites, such as aspartate required for cancer cell proliferation^10^.

Pancreatic ductal adenocarcinoma (PDAC) is the most aggressive cancer type worldwide^11^. In PDAC, both the Warburg and anti-Warburg effect have been reported. For example, activation of the KRAS proto-oncogene (*KRAS*) in PDAC cells has been shown to support cancer cell growth by enhancing the nucleotide biosynthesis through the PPP^12^. However, mitochondrial respiration sustained by high mobility group box protein 1 (HMGB1)^13^ and peroxisome proliferator-activated receptor gamma coactivator 1-alpha (PGC-1α)^14^ were found essential for PDAC tumor and PDAC cancer stem cell growth, respectively. Hence, the relationship between the development of PDAC and metabolic heterogeneity remains understudied.

Most patients with PDAC were diagnosed at a late stage and died within several months. One of the major reasons for the extreme aggressive properties of PDAC is the high metastatic potential of PDAC cells. Therefore, understanding the underlying mechanisms supporting the metastatic ability of PDAC cells might be the key in the therapeutic management of PDAC. As already mentioned, a number of studies showed that functional mitochondrial respiration was essential for cancer metastasis^9,15^. Consistently, our recent study on PDAC further revealed that mitochondrial respiration maintained by heat shock protein family D member 1 (HSPD1, also known as HSP60) was essential for the migration of PDAC cells^16^, suggesting that mitochondrial respiration might be positively associated with the metastasis of PDAC cells.

The mitochondrial oxidative phosphorylation system (OXPHOS) is comprised of five complexes which are the master regulators of cellular metabolism. However, both the regulation and metabolic contribution of OXPHOS in the growth and metastasis of PDAC cells remain unknown. In this study, we found that most OXPHOS encoding subunits were upregulated in PDAC tissues when compared with normal pancreatic tissues. Among these, upregulation of cytochrome c oxidase subunit 6B2 (*COX6B2*) was the most prominent. Noted, COX6B2 is an encoding subunit of the mitochondrial respiratory complex IV^17^, but its function in OXPHOS and human disease have not been explored. We set out to comprehensively evaluate the functional contribution of COX6B2 in PDAC tumorigenesis and uncovered a COX6B2/OXPHOS dependent role in the metastasis of PDAC cells.

## Results

### COX6B2 was increased in PDAC and associated with poor prognosis

**Fig. 1:**
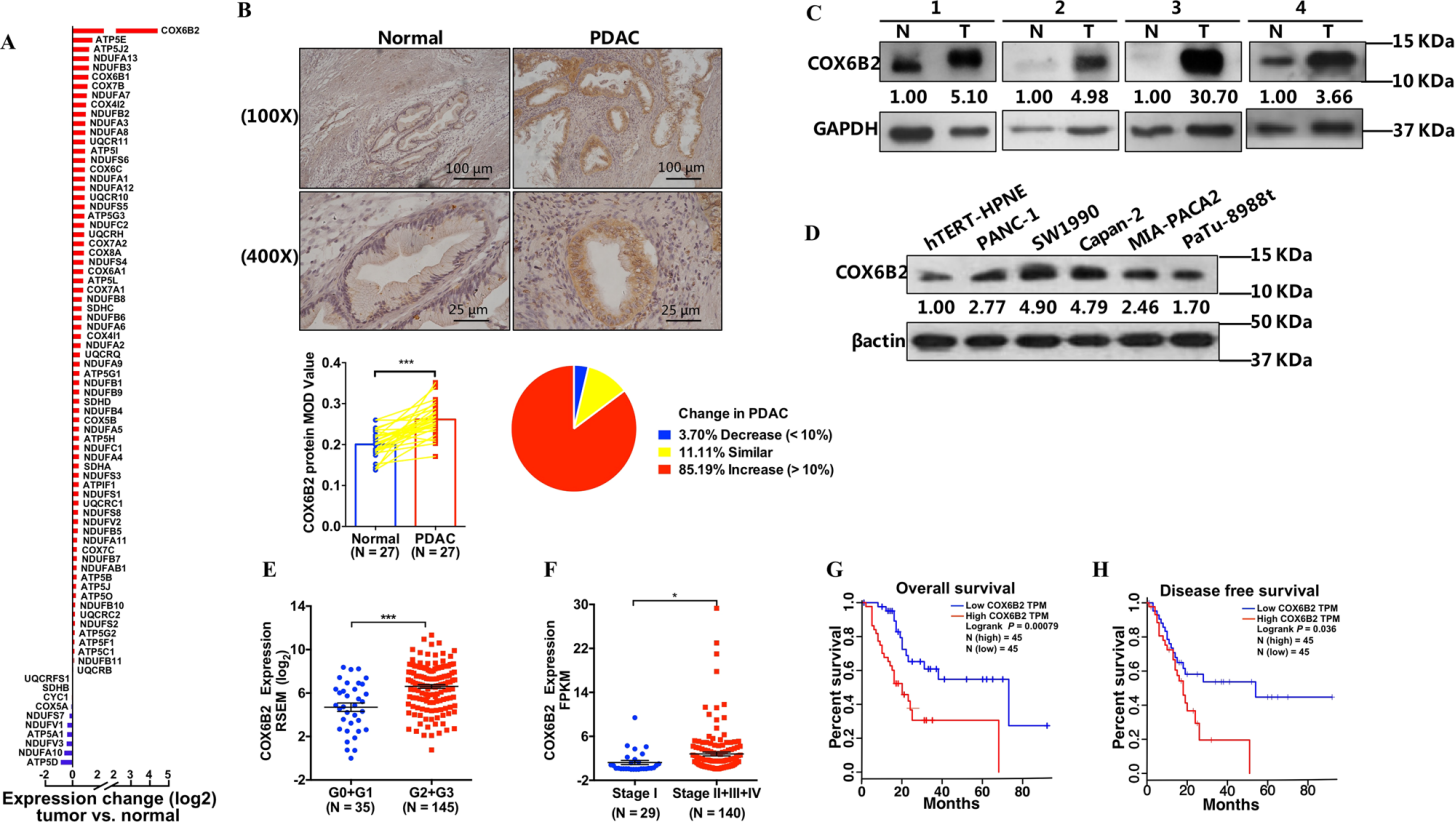
COX6B2 is increased in PDAC and associated with poor prognosis. **a** The bar plot shows the log_2_ (fold changes) of nuclear encoded OXPHOS genes between PDAC and normal tissues from TCGA and GTEx datasets, respectively. Red and blue bars indicate increase and decrease in gene expression, respectively. **b** Immunohistochemistry results of COX6B2 in PDAC tissues (*n* = 27) and matched adjacent normal tissues (*n* = 27). Representative immunohistochemistry photomicrographs (upper panel) at 100× (scale bar = 100 μm) and 400× (scale bar = 25 μm) magnification, quantitative results (lower left panel), and expression changes in PDAC tissues (lower right panel), respectively. **c** Western blot analysis of COX6B2 in fresh PDAC (T) and paired adjacent normal tissues (N). **d** Western blot analysis of COX6B2 in the HPNE (hTERT-HPNE) normal pancreatic ductal epithelial cell line and 5 PDAC cell lines. **e** Tumor samples are grouped by histological grade: G0 (*n* = 4), G1 (*n* = 31), G2 (*n* = 96), and G3 (*n* = 49). The column shows a dot map of the expression of *COX6B2* in PDAC with different histological grades: G0 + G1 compared with G2 + G3. **f** Comparison of *COX6B2* mRNA levels in PDAC tissues with (Stage II + III + IV, *n* = 140) or without (Stage I, *n* = 29) distance metastasis. **g**, **h** Overall (**g**) and disease-free (**h**) survival between patients with high (red) and low (blue) levels of *COX6B2* mRNA from the TCGA database (http://gepia.cancer-pku.cn). Patients with high and low levels of *COX6B2* were grouped with cut-off using quartile value. All data are presented as mean ± SEM (*n* ≥ 3). *P < 0.05, ***P < 0.001.

To nominate the possible involvement of OXPHOS subunits in the development of PDAC, we downloaded mRNA expression data of both patients and control subjects from databases of The Cancer Genome Atlas (TCGA) and the genotype-tissue expression (GTEx), respectively, and ranked OXPHOS encoding genes based on the expression differences observed between patients and controls. Among 81 nuclear encoding genes, the mRNA levels of 75 genes were significantly altered, of which most genes were upregulated in PDAC when compared with control tissues. Among all studied genes, the difference in the expression of *COX6B2* between PDAC and control tissues was ranked in the top (Fig. 1a, Fig. S1A). Consistently, protein analysis using paraffinized PDAC (Fig. 1b), fresh tissue samples (Fig. 1c), and cell lines (Fig. 1d) confirmed that the protein level of COX6B2 was significantly elevated in cancerous cells compared with normal cells. Moreover, we found that the mRNA level of *COX6B2* in PDAC tissues was top ranked among all 30 studied cancer types in the database of TCGA (Fig. S1B). Similarly, the mRNA level of *COX6B2* was more than tenfold greater in the PDAC cell line relative to any other cancer cell line from cancer cell line encyclopedia and was almost twofold greater than that in a lung cancer cell line (Fig. S1C)^18^. All these findings indicated that COX6B2 is a key feature of PDAC. Furthermore, combined analysis of the associations between the expression levels of *COX6B2* and the clinical outcomes of PDAC revealed that *COX6B2* mRNA was significantly increased in poorly differentiated compared with well differentiated PDAC cells (Fig. 1e), and in PDAC tissue with distant metastasis compared with nonmetastatic PDAC tissues (Fig. 1f). Probably as a result, patients with high levels of *COX6B2* would be bearing low percentage of overall and disease-free survival (Fig. 1g, h).

### Expression of *COX6B2* modulated the metastatic potential of PDAC cells

**Fig. 2:**
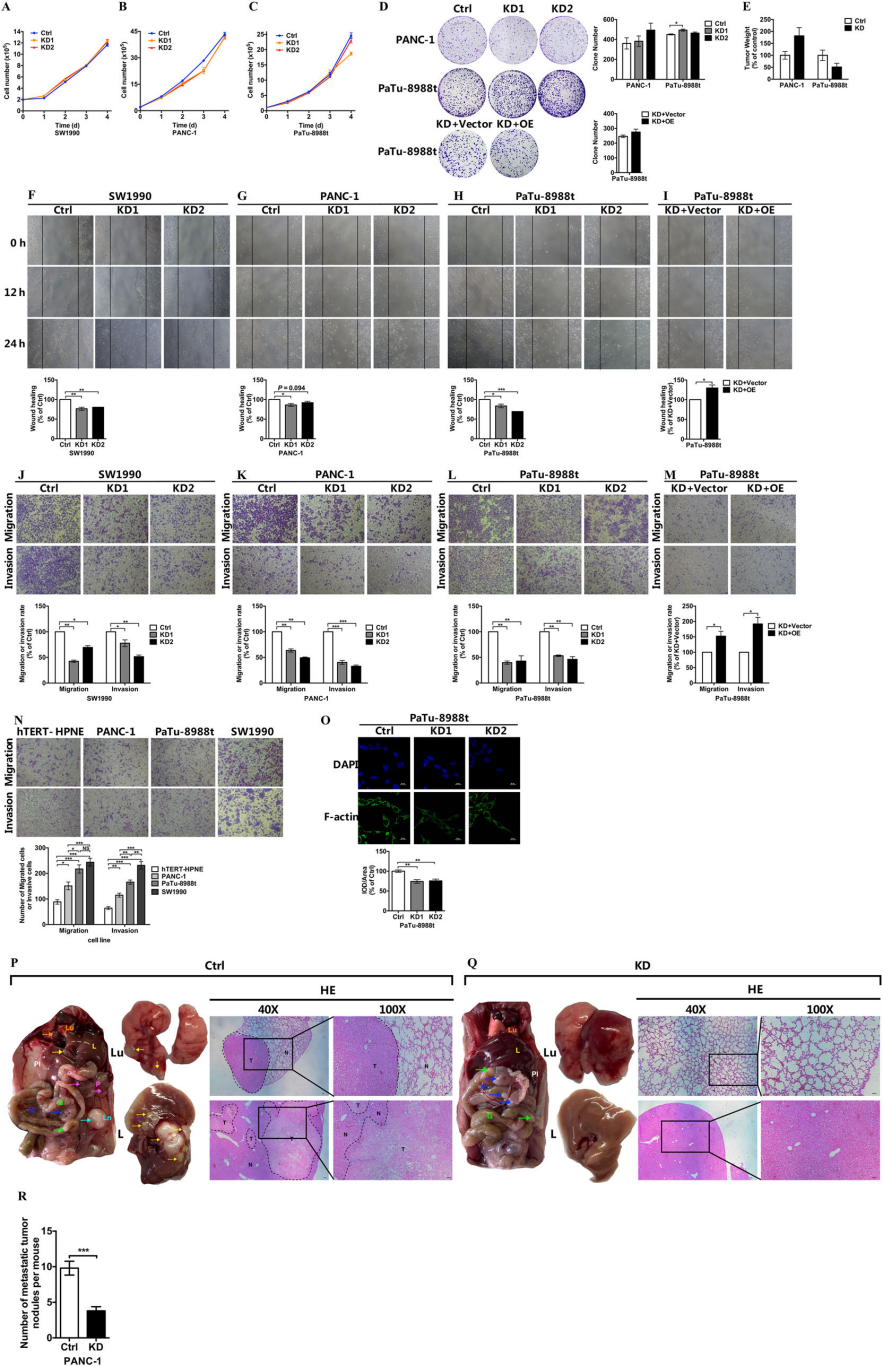
Expression of COX6B2 modulates metastatic potential of PDAC cells in vitro and in vivo. **a**–**c** Proliferation curves of three stable cell lines (SW1990 (**a**), PANC-1 (**b**), PaTu-8988t (**c**)) with knockdown (KD) of *COX6B2* compared with corresponding control (Ctrl) cells. **d** Colony formation assays of *COX6B2* KD PANC-1 (upper panel) and *COX6B2* KD PaTu-8988t (middle panel) cells, and cells with re-expression of *COX6B2* (lower panel) compared with corresponding control cells. Histograms on the right show the quantitative results, respectively. **e** Tumor xenograft experiments of PANC-1 and PaTu-8988t cells (5 × 10^6^ cells/mouse). All mice were sacrificed after 9 (PANC-1) or 8 (PaTu-8988t) weeks to measure the final tumor weight. (*n* = 6 mice per group). **f**–**i** Wound healing assays of 3 stable cell lines (SW1990 (**f**), PANC-1 (**g**), PaTu-8988t (**h**)) with knockdown of *COX6B2*, and of a *COX6B2* KD PaTu-8988t cell line with re-expression of *COX6B2* (**i**) compared with corresponding control cells. **j**–**m** Transwell assays show the migration (upper panel) and invasion (lower panel) abilities of three stable cell lines (SW1990 (**j**), PANC-1 (**k**), PaTu-8988t (**l**) with knockdown of *COX6B2*, and cell line with re-expression of *COX6B2* (**m**). **n** Transwell assays of the HPNE (hTERT-HPNE) normal pancreatic ductal epithelial cell line and five PDAC cell lines as indicated. **o** Representative immunofluorescence photomicrographs of *COX6B2* KD PaTu-8988t cells compared with control cells. Photomicrographs were captured using a confocal laser microscope (600× magnification, scale bar = 25 μm). Actin cytoskeleton and nuclei were stained with anti-F-actin (green) and DAPI (blue), respectively. **p**, **q** Representative images of macro-anatomy (left panel), lung (Lu) (middle upper panel), liver (L) (middle lower panel), and paired H&E-stained images after tail vein injection with control PANC-1 cells (**p**) and PANC-1 cells with KD of *COX6B2* (**q**). For 40× magnification, scale bar = 50 μm; For 100× magnification, scale bar = 20 μm. Arrows indicate metastatic sites in abdominal cavity: gut (G), mesentery (M), pancreatic (P), lymph nodes (Ln), and pleura (Pl). T tumor, N adjacent normal tissue. *n* = 5 mice per group. **r** Average number of metastatic tumor nodules in mouse after tail vein injection with control PANC-1 cells and PANC-1 cells with KD of *COX6B2*. *n* = 5 mice per group. Histograms show the quantitative results respectively. All data are presented as mean ± SEM (*n* ≥ 3). *P < 0.05, **P < 0.01 and ***P < 0.001. NS no significance.

To uncover the impact of COX6B2 on PDAC cells, we generated *COX6B2* knockdown (KD) stable cell lines in SW1990, PANC-1, and PaTu-8988t cells (named 8988 hereafter) (Fig. S2A–C). In addition, we further performed re-expression of *COX6B2* in *COX6B2* KD 8988 cells (Fig. S2D, E). We found that suppression of *COX6B2* did not affect cancer cell growth in all three studied cancer cell lines (Fig. 2a–c). Both the in vitro (Fig. 2d) and in vivo (Fig. 2e) tumor formation assays in PANC-1 and 8988 cells further confirmed that modulating the expression level of *COX6B2* had no effect on tumor formation. The tumor formation assay performed in SW1990 cells was not presented due to the difficulty in forming a clone and tumor. Although, *COX6B2* KD in all three studied cancer cell lines inhibited the migration of PDAC cells (Fig. 2f–h) in the performed wound healing assays, re-expression of *COX6B2* in *COX6B2* KD 8988 cells restored their migration ability (Fig. 2i). The effect of *COX6B2* on the metastatic potential of PDAC cells was much more significant when using the transwell assay, a commonly used assay to test the migratory ability of cancer cells. As shown in Fig. 2j–l, all three *COX6B2* KD PDAC cell lines showed a significant decrease of invasion and migration ability, whereas overexpression of *COX6B2* resulted in their increased invasion and migration ability (Fig. 2m). Consistently, PDAC cell lines with higher levels of *COX6B2* (Fig. 1d) exhibited increased invasion and migration ability compared with cell lines with lower levels of *COX6B2* (Fig. 2n). Furthermore, *COX6B2* KD cells had lower levels of filamentous actin (F-actin) (Fig. 2o). Moreover, overexpression of COX6B2 in PANC-1 and 8988 cells (Fig. S3A, B), which have lower level of COX6B2 than SW1990 (Fig. 1d), significantly increased the migration ability of PDAC cell (Fig. S3C, D). Consistently, in vivo metastatic experiments revealed that KD of *COX6B2* in PANC-1 cells were associated with significantly lower ability to generate lung and liver metastasis (0/5 mice for lung; 1/5 mice for liver) when compared with control cells in nude mice (5/5 mice for lung; 5/5 mice for liver) (Fig. 2p, q). Furthermore, the number of surface nodules in abdominal cavity were significantly lower in mice with *COX6B2* KD PANC-1 cells than mice with control PANC-1 cells (Fig. 2r). Altogether, these results indicated that COX6B2 can modify the metastatic potential of PDAC cells without affecting cancer cell growth and tumor formation.

### COX6B2 facilitated the assembly of complex IV

**Fig. 3:**
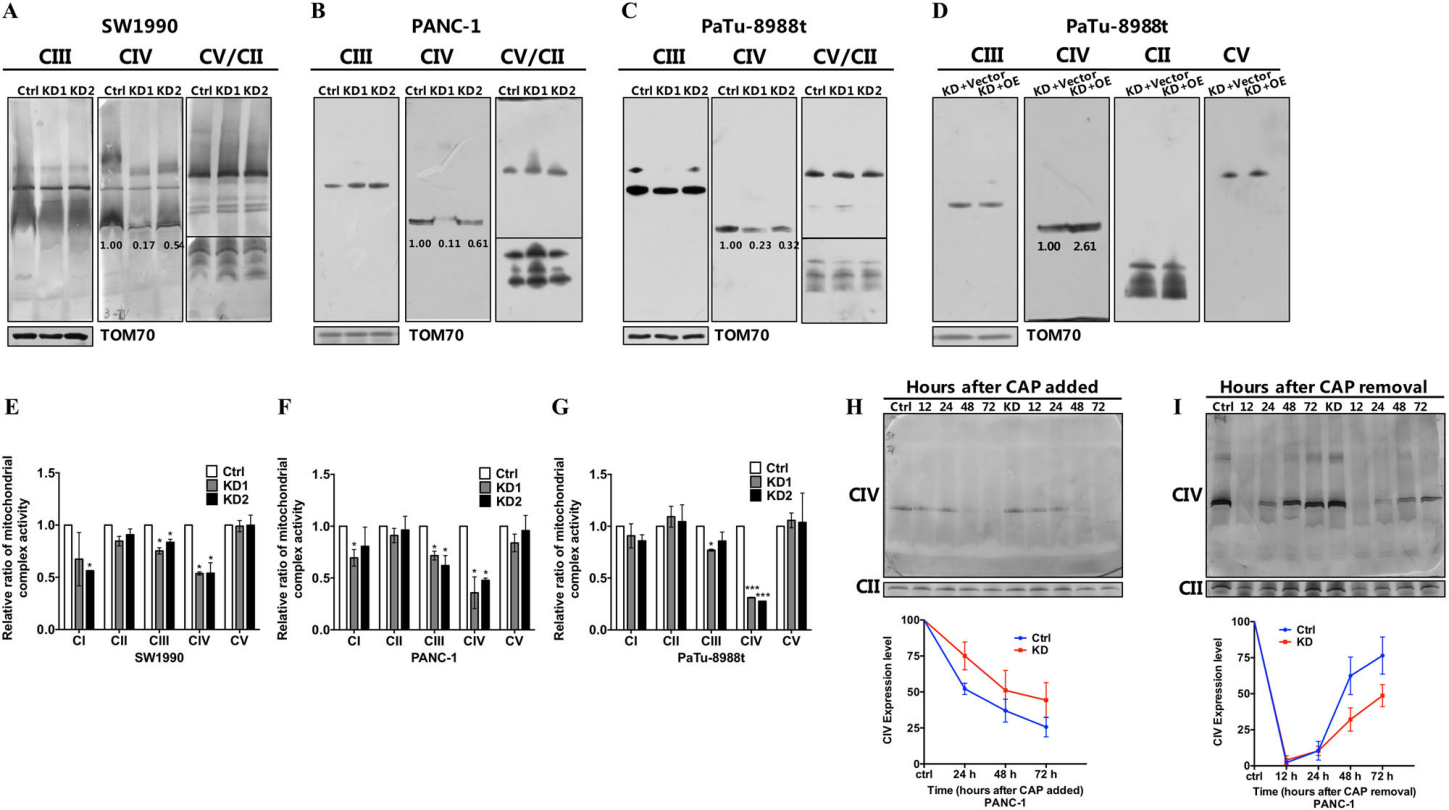
COX6B2 facilitates the assembly of complex IV. **a**–**d** 2 % DDM-permeabilized cells analyzed by BNG, illustrate mitochondria monomer complexes separated in SW1990 (**a**), PANC-1 (**b**), and PaTu-8988t (**c**) cells with knockdown of *COX6B2* and *COX6B2* re-expression cell line (**d**). Blots were probed with anti-UQCRC2, anti-COI, anti-SDHA, and anti-ATP5A for complex III (CIII), complex IV (CIV), complex II (CII), and complex V (CV), respectively. **e**–**g** The activity of OXPHOS complexes (complex I–V) was measured with mitochondria isolated from SW1990, PANC-1, PaTu-8988t cells with knockdown of *COX6B2* and paired control cells. Data are normalized to the activity of citrate synthase and the activity of control cells was taken as 100%. **h**, **i** The ability of mitochondrial monomer IV degradation (**h**) and assembling (**i**) in *COX6B2* KD PANC-1 cells is compared to control cells. To analyze the degradation ability of monomer IV, cells were treated with 40 μg/mL chloramphenicol (CAP) and cell pellets were collected after CAP was added at 24, 48, and 72 h. To analyze the assembling ability of monomer IV, cells were treated with CAP for 7 d, and cell pellets were collected after CAP removal at 12, 24, 48, and 72 h. Blots were probed with anti-MT-COI, anti-SDHA for complex IV (CIV) and complex II (CII) using 2% DDM-permeabilized cells, respectively. Ctrl: cells without treatment of CAP. All data are presented as mean ± SEM (*n* ≥ 3). *P < 0.05, ***P < 0.001.

Noted, COX6B2 was first identified as an isoform of COX6B1, a subunit of mitochondrial complex IV with unknown function, localized in testis^17^. To ask whether the regulation of the metastatic potential of PDAC cells by COX6B2 involved a mitochondrial specific role, we tested the effect of *COX6B2* on mitochondrial complex IV assembly in three PDAC cell lines. As shown in Fig. 3a–c, suppression of *COX6B2* in SW1990, PANC-1, and 8988 cells led to the decrease of monomer complex IV by 33–55%, whereas rescued expression of *COX6B2* restored the steady state levels of complex IV (Fig. 3d). Analysis of the enzymatic activity of all five OXPHOS complexes revealed that knockdown of *COX6B2* in PDAC cell lines significantly lowered the activity of complex IV (Fig. 3e–g). Although we did observe a minor decrease in the activity of complex I and III in some but not all *COX6B2* KD cells when compared with control cells, all other OXPHOS complexes remained unaffected (Fig. 3e–g). These results indicated that knockdown of *COX6B2* was specifically associated with complex IV. Since either the defect of assembly or decrease of stability of complex IV can lead to the decreased levels of activity of complex IV, we next sought to distinguish between these scenarios by performing assembly and degradation kinetics of complex IV in *COX6B2* KD cells. To follow the degradation of mitochondrial complex IV in 8988 cells, we first treated cells with the reversible mitochondrial translation inhibitor chloramphenicol (CAP) to block assembly of OXPHOS complexes by newly synthesized subunits. Cells were cultured with CAP for as long as 72 h and collected at various time points for blue native gel (BNG) analysis. However, no difference was observed on the kinetics of complex IV degradation between *COX6B2* KD cells and control cells (Fig. 3h). We then took an alternative approach and sought to follow the assembly process of complex IV. Cells were cultured in the presence of CAP for 7 d to block the synthesis of new mtDNA-encoded subunits and thus exhaust the existing OXPHOS complexes. To profile the assembly of new OXPHOS complexes, we collected cells at various time points after CAP removal and analyzed the respiratory complexes by BNG analysis. As shown in Fig. 3i, assembling of complex IV was slower in *COX6B2* repressed than in control cells. To further understand the role of COX6B2 on the assembly of complex IV, we also generated and examined a 293T cell line with depletion of *COX6B2* (Fig. S4A). Similarly, 293 cells lacking *COX6B2* had lower levels of fully assembled monomer complex IV relative to control cells (Fig. S4B). Moreover, using high resolution BNG analysis revealed accumulations of large sub-complexes IV in *COX6B2* null cells (Fig. S4C), suggesting that COX6B2 facilitates the assemble of complex IV at late stage of complex IV assembly. Of note, all complex IV containing supercomplexes, including complex I + III + IV, complex III + IV, and dimerized forms of complex IV, were decreased in 293 cells lacking COX6B2 when compared with control cells (Fig. S4D). Taken together, these findings suggested that *COX6B2* facilitated the assembly of monomer complex IV, and loss of COX6B2 led to the defective assembly of all complex IV containing supercomplexes.

### COX6B2 repression inhibited the function of oxidative phosphorylation without boosting Warburg effect

**Fig. 4:**
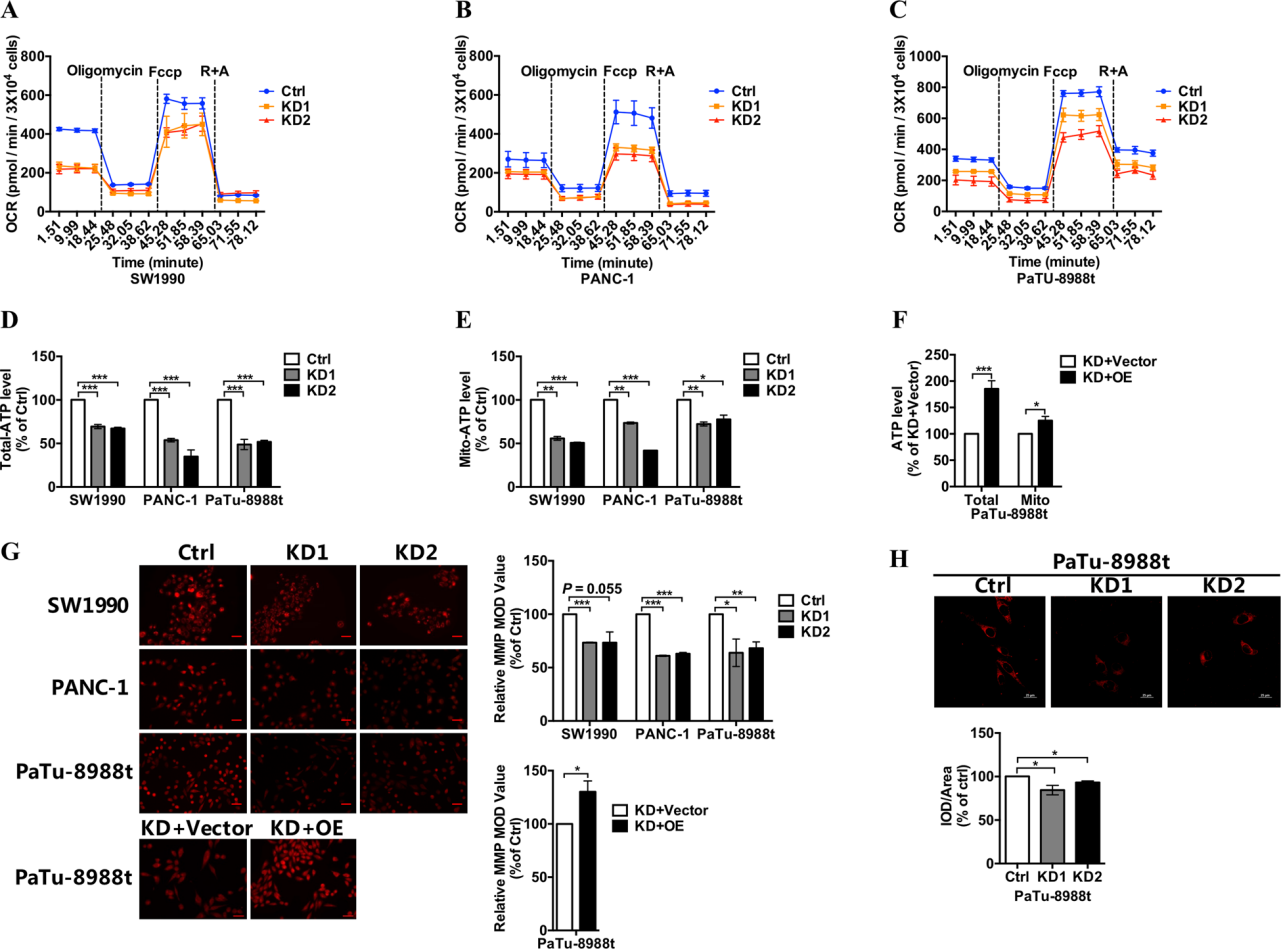
Loss of COX6B2 further reduces oxygen consumption, ATP levels, and MMP levels. **a**–**c** Oxygen consumption rate (OCR) of SW1990, PANC-1, PaTu-8988t cells with knockdown of *COX6B2* and paired control cells using the Seahorse XF24 flux analyzer after successive addition of oligomycin (1 μM), FCCP (0.5 μM), and rotenone/antimycin A (0.5 μM/0.5 μM). **d** Total intracellular ATP levels in cultured SW1990, PANC-1, PaTu-8988t cells with knockdown of *COX6B2* and paired control cells. Total intracellular ATP was measured using bioluminescence assays. **e** Mitochondrial ATP levels in SW1990, PANC-1, PaTu-8988t cells with knockdown of *COX6B2* and paired control cells. Mitochondrial ATP was measured using bioluminescence assays. **f** Total intracellular ATP and mitochondrial ATP levels of *COX6B2* KD PaTu-8988t cells with re-expression of *COX6B2* and paired control cells. **g** MMP levels of SW1990 (first panel), PANC-1 (second panel), PaTu-8988t (third panel) cells with knockdown of *COX6B2* and re-expression of *COX6B2 in* PaTu-8988t cell with knockdown of *COX6B2* (fourth panel) compared with corresponding control cells. Representative photomicrographs were captured using a fluorescence microscope (400× magnification, scale bar = 25 μM). **h** Representative fluorescence photomicrographs captured using a confocal laser microscope (600× magnification, scale bar = 25 μM) in *COX6B2* KD PaTu-8988t cells compared with control cells. Cells were stained with 2 μM BODIPY 581/591 C11 in serum-free DMEM for 30 min. All data are presented as mean ± SEM (*n* ≥ 3). *P < 0.05, **P < 0.01, and ***P < 0.001.

Based on our findings, we considered that COX6B2 might play an essential role in the regulation of the metastatic potential of PDAC cells by changing the function of OXPHOS complexes. We evaluated the mitochondrial function of OXPHOS by measuring mitochondrial respiration and found that *COX6B2* KD PDAC cells exhibited decreased endogenous mitochondrial respiration (Fig. 4a–c). Moreover, both cellular and mitochondrial derived ATP production were lower in *COX6B2* repressed cells than in control cells (Fig. 4d, e), whereas rescued expression of *COX6B2* in *COX6B2* repressed 8988 cells restored ATP generation (Fig. 4f). Consistently, PANC-1 and 8988 cells with overexpression of COX6B2 have higher cellular ATP production than control cells (Fig. S5A, B). Accordingly, decreased mitochondrial membrane potential (MMP) was detected in *COX6B2* KD PDAC cells compared with control cells, whereas rescued expression of *COX6B2* in *COX6B2* KD cells restored MMP (Fig. 4g). Notably, transcriptome analysis revealed that mitochondrial transcriptional products and mitochondrial bioenergetics markers including *RXRA*, *PGC1α*, and *NRF2* were not differed between control 8988 cells and 8988 cells with KD of *COX6B2* (Fig. S6A, B), suggesting that COX6B2 regulates the function of OXPHOS without affecting the regulatory mechanisms of mitochondrial biogenesis.

Impaired mitochondrial respiration has been reported as the key driver force in cancer development by inducing the Warburg effect^19^, which is opposite to our findings on *COX6B2* repression in PDAC cells. We agreed that knockdown of *COX6B2* resulted in downregulated mitochondrial respiration; however, a boosted Warburg effect was not observed in *COX6B2* KD cells, while neither cell nor tumor growth were altered by the repression of *COX6B2* (Fig. 2a–e), which was further confirmed by the fact that extracellular acidification rate was not altered in 8988 cells with KD of *COX6B2* (Fig. S7). Furthermore, gene set enrichment analysis (GSEA) of *COX6B2* KD 8988 cells did not show any significant enrichment of transcriptional alterations related to metabolic processes, including glycolysis and the PPP (Table S1). Concurrently, although 8988 cells with different expression levels of *COX6B2* showed distinct profiles of metabolic features (Fig. S8A), metabolic profiling further revealed that both glycolysis (Fig. S8B) and the lipid levels (Fig. 4h) were more likely to be repressed rather than boosted in *COX6B2* KD cells with inhibited OXPHOS complexes. Moreover, 14 and 6 pyrimidine and purine metabolites were downregulated and upregulated, respectively, in *COX6B2* repressed 8988 compared with control cells (Fig. S8C, D), indicating that at least the anabolic features were not favored in *COX6B2* KD cells. Concomitantly, most amino acid metabolites were downregulated in *COX6B2* KD 8988 compared with control cells (Fig. S8E). Among these, aspartate, an amino acid essential for cell growth^20^, was downregulated by 40%. In addition, inhibition of citric acid cycle (TCA) (Fig. S8F) in *COX6B2* KD 8988 cells might have also contributed to the decreased levels of amino acids (Fig. S8E). Collectively, these results further demonstrated that the high levels of COX6B2 in PDAC were more likely to promote the metastatic ability of PDAC cells and had little effect on cancer cell growth and tumor formation.

### COX6B2 promoted the metastasis of PDAC cells via the ATP/purinergic receptor pathway

**Fig. 5:**
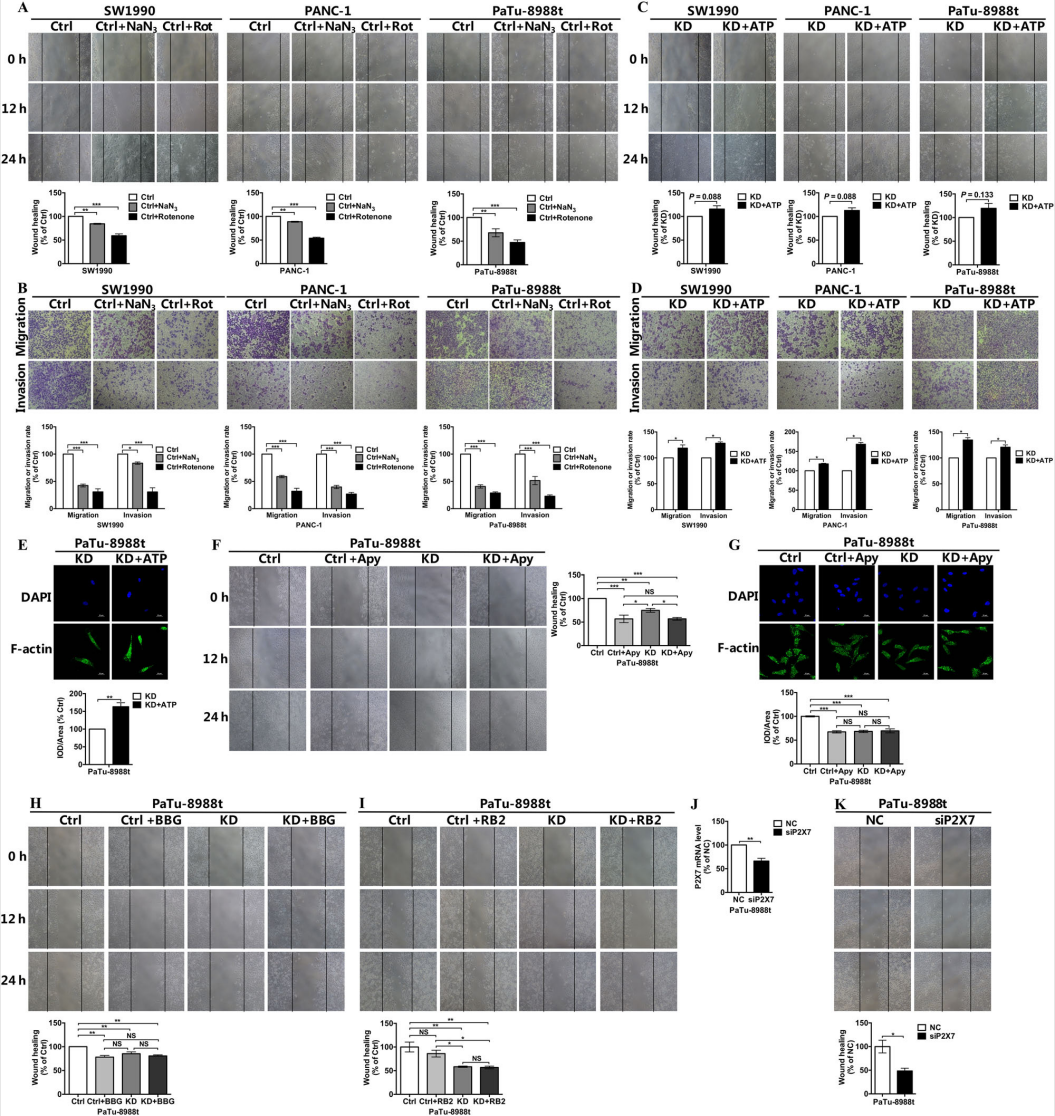
COX6B2 promotes PDAC cells metastasis via ATP/purinergic receptor pathway. **a**, **b** The migration and invasion ability of SW1990, PANC-1, and PaTu-8988t cells with or without NaN_3_ (50 μM) or rotenone (200 nM) was evaluated using wound healing assays (**a**) and trans-well assays (**b**). **c**, **d** The migration and invasion ability of SW1990, PANC-1, and PaTu-8988t cells with knockdown of *COX6B2* with or without ATP (100 μM) was evaluated using wound healing assays (**c**) and transwell assays (**d**). **e** Representative immunofluorescence photomicrographs captured using a confocal laser microscope (600× magnification, scale bar = 25 μm), *COX6B2* KD PaTu-8988t cells with or without ATP (100 μM) were probed with F-actin (1:100) and a fluorescently labeled IgG-Alexa Fluor 488 secondary antibody (1:500). **f** Wound healing assays of *COX6B2* KD PaTu-8988t cells and paired control cells with or without apyrase (2.5 mU/mL). **g** Representative immunofluorescence photomicrographs of *COX6B2* KD PaTu-8988t cells and paired control cells with or without apyrase (2.5 mU/mL). Cells were probed with anti-F-actin (1:100) and a fluorescently labeled IgG-Alexa Fluor 488 secondary antibody (1:500). Photomicrographs were captured using a confocal laser microscope (600× magnification, scale bar = 25 μm). **h**, **i** Wound healing assays of *COX6B2* KD PaTu-8988t cells and paired control cells with or without BBG (10 μM) or RB2 (10 μM). **j** qRT-PCR analysis demonstrates the knockdown efficiency of *P2X7* in PaTu-8988t cells. **k** Wound-healing assays of *P2X7* KD PaTu-8988t cells. All data are presented as mean ± SEM (*n* ≥ 3). *P < 0.05, **P < 0.01, and ***P < 0.001. NS no significance.

To investigate whether *COX6B2* promoted metastasis of PDAC cells via inhibition of OXPHOS activity, the function of OXPHOS complexes was inhibited using the rotenone and NaN_3_, inhibitors of OXPHOS complex I and complex IV, respectively. Both the wound healing (Fig. 5a) and transwell assays (Fig. 5b) revealed that inhibition of OXPHOS led to the suppression of the invasion and migration ability of PDAC cells. Mechanistically, modulation of the function of mitochondrial OXPHOS has been shown to lead to the alteration of mitochondrial-nuclear cross talks mediated by changes in the levels of mitochondria-to-nucleus retrograde signaling mediators, such as calcium, reactive oxygen species (ROS), and AMP^21,22^. Although all three *COX6B2* KD PDAC cancer cell lines were found to exhibit altered mitochondrial signals, including increased levels of ROS and AMP (Fig. S9A–D), as well as mitochondrial calcium overload (Fig. S9E), unexpectedly, elimination of ROS and inhibition of AMP-activated protein kinase (AMPK) pathways did not increase but rather decreased the metastatic potential of PDAC cells (Fig. S9F, G). Moreover, since mitochondrial calcium overload gas been shown to function as an inducer of cell migration, its inhibition might suppress and not promote migration of PDAC cells^23^. Thus, it could be inferred that metastasis of PDAC cells regulated by *COX6B2* and the function of OXPHOS complexes might not be mediated by classic mitochondrial to nuclear signaling pathways.

Because decreased mitochondrial ATP has been revealed to be a direct consequence of *COX6B2* repression in PDAC cells, we next aimed to determine whether ATP availability plays a role in cell metastasis. Both the wound healing (Fig. 5c) and transwell assays (Fig. 5d) revealed that exogenous ATP complementation in *COX6B2* KD PDAC cells partially restore the migratory ability of cells. In agreement with the migration assay, *COX6B2* KD PDAC cells had lower levels of F-actin than cells supplemented with ATP (Fig. 5e). This result indicated that *COX6B2* might enhance generation of mitochondrial ATP by incrementing the function of OXPHOS to support the metastasis of cancer cells. Notably, cleavage of extracellular ATP by apyrase lowered the migratory ability of control 8988 cells, comparable to the *COX6B2* KD cells (Fig. 5f). However, apyrase had little effect on the migratory ability of *COX6B2* KD 8988 cells (Fig. 5f). Similarly, apyrase supplementation decreased F-actin levels in control 8988 cells, whereas *COX6B2* KD cells remained unaffected (Fig. 5g). Furthermore, inhibition of the purinergic receptor P2X5/7 by brilliant blue G (BBG) (Fig. 5h), but not that of P2Y by reactive blue 2 (RB2) (Fig. 5i) mimicked the effect of ATP cleavage by apyrase. Consistently, knockdown of P2X7 in 8988 cells led to decreased migration ability compared with control cells (Fig. 5j, k). Collectively, these results demonstrated that COX6B2 repression reduced OXPHOS derived ATP generation to inhibit metastasis of PDAC cells via purinergic receptor pathway.

### Metformin suppressed the metastasis of PDAC cells by downregulating the levels of COX6B

**Fig. 6:**
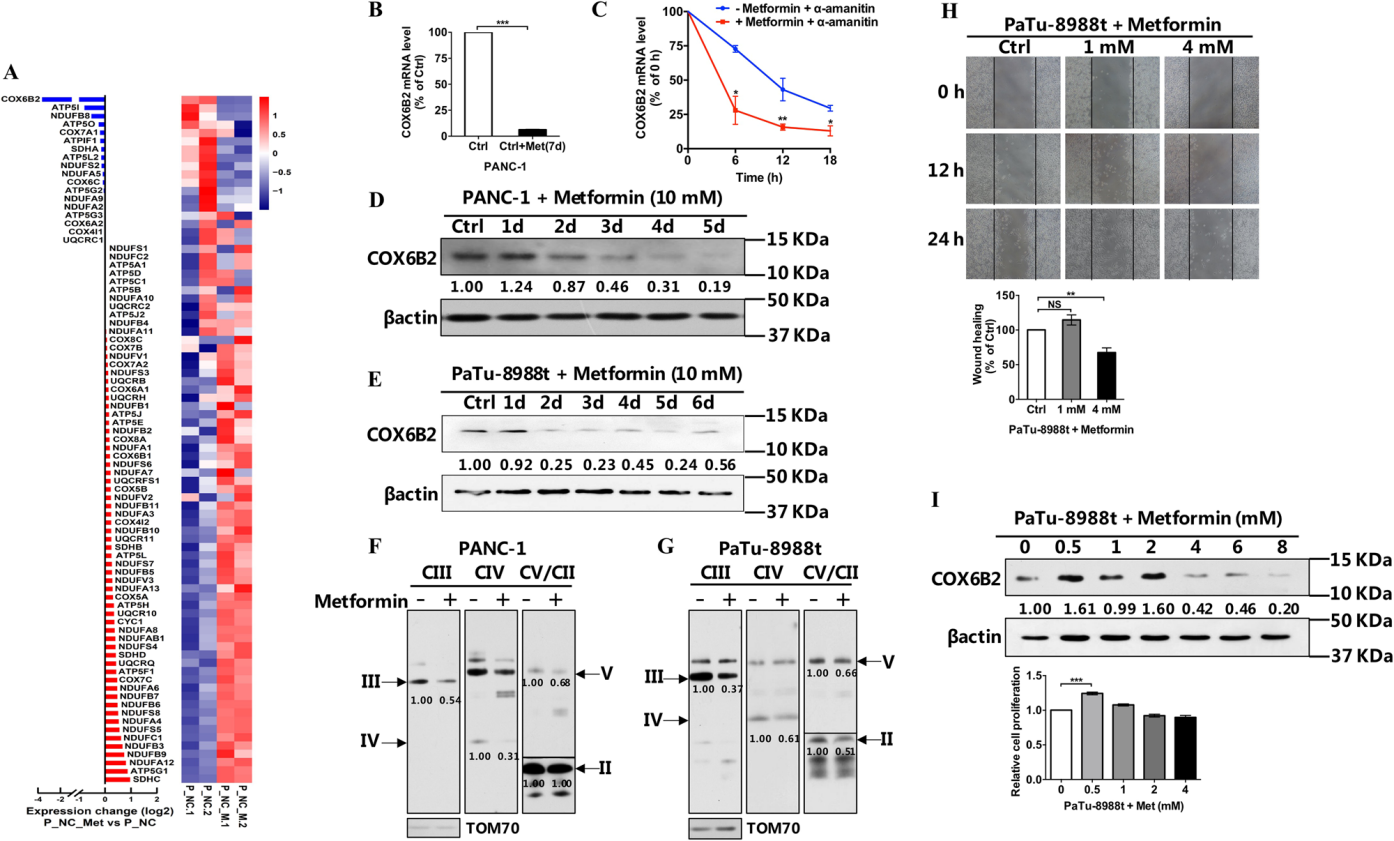
Metformin suppress metastasis of PDAC cells. **a** Transcriptional analysis of the nuclear encoded OXPHOS genes of PDAC cells treated with metformin and of the untreated control cells. Left side: bar plot of the fold change of genes between two groups. Red bars indicate increase and blue bars decrease in gene expression. Right side: heatmap of the expression *z*-scores of the genes among samples. **b** Effects of metformin on the expression of *COX6B2* mRNA. qRT-PCR analysis provides the *COX6B2* mRNA levels in PANC-1 cells after treating with metformin for 7 d. This experiment was performed in technical triplicates. **c** qRT-PCR analysis of *COX6B2* mRNA levels in PANC-1 cells with or without metformin (10 mM). The time point for the culture of cells with α-amanitin (50 μM) were indicated. Before the addition of α-amanitin, cells were pretreated with or without metformin (10 mM) for additional 36 h. The experiment was performed in triplicates and was independently repeated (*n* = 2). **d**, **e** Western blot analysis of COX6B2 in PANC-1 (**d**) and PaTu-8988t (**e**) cells after metformin (10 mM) treatment for 1–6 d. **f**, **g** 2% DDM-permeabilized PANC-1 cells (**f**) and 8988 cells (**g**) analyzed by BNG show mitochondrial monomer complex II–V levels after metformin (10 mM) treatment for 2 days. Cells without metformin were used as control. Complex II–V were probed with anti-SDHA, anti-UQCRCR, anti-COI, and anti-ATP5A, respectively. **h** Wound healing assays of PaTu-8988t cells after treatment with various concentrations (0, 1, and 4 mM) of metformin. **i** Western blot analysis of COX6B2 (upper panel) and cell proliferation assays (lower panel) of PaTu-8988t cells after treatment with various concentrations (0, 0.5, 1, 2, 4, 6, and 8 mM) of metformin. All quantified data are presented as mean ± SEM (*n* ≥ 3). *P < 0.05, **P < 0.01, ***P < 0.001. NS no significance.

Metformin has been reported as a therapeutic drug used to treat PDAC^16^, but its role in PDAC remains unknown. Transcriptional analysis of PDAC cells treated with metformin revealed that the alteration of the expression of *COX6B2* was ranked at the top among all nuclear encoded OXPHOS genes (Fig. 6a, b). Moreover, by inhibiting the RNA transcription with α-amanitin in PANC-1 cells, we found that degradation of *COX6B2* mRNA was faster in cells with metformin than control cells (Fig. 6c). This result indicates that metformin represses the expression of *COX6B2* by promoting the degradation of *COX6B2* mRNA. Consistently, both PANC-1 and 8988 cells treated with metformin showed decreased levels of *COX6B2* in a time dependent manner (Fig. 6d, e). BNG analysis revealed that metformin treated PANC-1 and 8988 cells exhibited lower levels of multi-OXPHOS complexes than untreated cells (Fig. 6f, g). Furthermore, we found that treatment of 8988 cells for 3 d with 4 mM metformin was sufficient to lead to a dramatic decrease in the levels of COX6B2, and to significantly inhibit their migratory ability (Fig. 6h), whereas cell replication remained unaffected (Fig. 6i). Altogether, our results suggested that metformin could mimic the effect of the *COX6B2* knockdown on PDAC cells by repressing the expression of *COX6B2*. Thus, metformin could potentially stand as an attractive drug in therapeutic approaches targeting cancer cells with high levels of *COX6B2*.

## Discussion

Metabolic reprogramming characterized by increased glycolysis and decreased function of OXPHOS complexes, being the key point of the Warburg effect, has been well documented in many cell types. However, such kind of metabolic requirements have now been challenged in many types of cancer, including PDAC^24^. The increased function of OXPHOS was found to be favored in PDAC cancer stem cells but not in PDAC cell lines^14,25,26^. Therefore, uncovering the underlying mechanisms by which OXPHOS regulate the development of PDAC could be of potential therapeutic value. In this study, we showed that the *COX6B2*-regulated function of OXPHOS was closely associated with the metastatic potential of PDAC cells. Mechanistically, it was demonstrated that COX6B2 facilitated the assembly of OXPHOS complex IV to support mitochondrial respiration and OXPHOS-induced generation of ATP. Notably, cells with repressed OXPHOS function due to KD of *COX6B2* appeared to exert little effect on the glycolytic process, and cancer cell growth both in vivo and in vitro, indicating that Warburg effect was not activated in PDAC cells with KD of *COX6B2*. Alternatively, COX6B2 might support the TCA outputs of amino acids for biosynthesis, which are essential for cell proliferation and cycle progression^27^. Although more studies are required to clarify the metabolic contributions of COX6B2 on PDAC, our obtained results, demonstrated that COX6B2 enhanced the function of OXPHOS complexes to promote metastasis of PDAC cells without compromising their anabolic features.

The *COX6B2* gene is a testes-specific isoform of *COX6B1*^17^. Ideally, COX6B2 might function like COX6B1 in connecting 2 monomers complex IV into a dimerized form. So far, the only known biological function attributed to *COX6B2* came from a study where knockdown of its expression led to the activation of apoptosis^28^; however, GSEA did not shown any significant enrichment of apoptotic pathways and cell proliferation was not significantly altered in *COX6B2* KD compared with control cells (Table S1, Fig. 2a–c), suggesting that its biological role might be cell type specific. The role of COX6B1/2 on the assembly of complex IV remains largely unknown. In yeast, depletion of *COX6B* had little effect on the assembly of complex IV but resulted in a dramatic decrease of its activity^29^. Unlike yeast, removal of *COX6B* from bovine heart cells led to increased activity of complex IV^30^, probably due to the fact that for the dimerization of complex IV COX6B was shown to bind to the active center of the monomer complex IV^31^. Moreover, it has been suggested that COX6B might most likely not be essential for the assembly and function of complex IV in plants^32^. All these results have indicated that the function of *COX6B* might be diversified and species specific. In human cells, COX6B1 was found to be essential for both the assembly and activity of complex IV^33^. In our study, we found that high levels of COX6B2 facilitated assembly rather than stabilization of the monomer complex IV and enhanced mitochondrial function in PDAC cells. While the only change in PDAC cells with varying levels of COX6B2 was the ratio of COX6B1/COX6B2, we proposed that there might be a competitive role between COX6B1 and COX6B2 in late stage assembly of complex IV, and COX6B2 might be more efficient in driving the assembly of complex IV compared with COX6B1. However, more studies are needed for further validation of the different kinetics of the assembly of complex IV between COX6B1 null and COX6B2 null cells.

We concluded that COX6B2 modulated the metastasis of PDAC cells through the regulation of the function of OXPHOS complexes, based on the fact that PDAC cells with high levels of COX6B2 were shown to enhance the function of OXPHOS by promoting the assembly of complex IV and also increase the metastatic potential of PDAC cells, whereas inhibition of OXPHOS led to decreased metastasis of PDAC cells. Changes in the function of OXPHOS complexes have been demonstrated to result in dramatic alterations of the mitochondria-to-nucleus retrograde signaling pathways^21^. Both the suppressed and enhanced function of OXPHOS were found to be associated with the ability for metastasis of PDAC cells^34,35^. In models of inhibition of OXPHOS, the resulting increased mitochondrial oxidative stress was shown to be the major driver force supporting cancer cell metastasis^34^. Although amelioration of oxidative stress did practically suppress the metastatic potential of PDAC cells in our models, we observed that *COX6B2* KD PDAC cells exhibited lower metastatic potential but still accumulated higher oxidative stress than control cells (Fig. S9A–G), suggesting that COX6B2 exerts an additional role in COX6B2 modulated PDAC cell metastasis. Moreover, because most cancer cells have an increased ROS detoxification system to keep cells away from apoptosis, we believe that the increased oxidative stress noted here was not the likely major driver force in the development of advanced stage cancer. Alternatively, we considered that the ATP generated by the activity of OXPHOS was the key driving the metastasis of PDAC cells. These ATP molecules were secreted in the extracellular matrix and stimulated the purinergic receptor pathway, a pathway known in positive regulation of epithelial–mesenchymal transition (EMT) and metastasis^36^. Our findings regarding the role of ATP on the metastasis of PDAC cells is consistent with a previous study on the metastasis of PGC-1α and breast cancer cells^15^, as well as with our previous report on the role of HSP60 in the tumorigenesis of PDAC^16^. Notably, the ATP/purinergic receptor pathway might be the most fitting major pathway candidate to drive COX6B2 modulated metastasis of PDAC cells, because inhibition of the pathway was sufficient to reverse the metastasis of PDAC cells induced by the expression of *COX6B2*. Meaningfully, our study is the first to provide a link between the function of OXPHOS and the activation of the purinergic receptor pathway. Based on this finding, and due to the observed metabolic heterogeneity of cancer cells in tumors, we speculated that EMT and cell metastasis could be acquired in cells with lower levels of activity of OXPHOS from cells with high levels. In addition, the tumor microenvironment, such as cancer-associated macrophages and platelets^37^, with highly activated function of OXPHOS complexes might also contribute to cancer cell metastasis via the OXPHOS/ATP/purinergic receptor axis. Furthermore, in addition to the known function of metformin on the function of complex I and cancer cell metastasis^38^, we revealed a novel role of metformin in targeting the function of OXPHOS by promoting the degradation of *COX6B2* mRNA. Clinical administration of metformin could pose as a new therapeutic strategy in patients with increased levels of *COX6B2*.

In summary, our findings demonstrated that *COX6B2* regulated the metabolic shift and facilitated metastasis in PDAC cells. Administration of metformin was revealed to be able to inactivate the COX6B2 mediated OXPHOS/ATP/purinergic receptor pathway and inhibit cancer cell metastasis.

## Materials and methods

### Cell lines and culture conditions

The SW1990, PANC-1, and 8988 pancreatic cancer cell lines were ordered from the Chinese Academy of Sciences (Shanghai, China). Identities of cell lines were authenticated based on analysis of short-tandem repeat loci (Genetic Testing Biotechnology Corporation, Suzhou, Jiangsu, China). All cells were cultured in high-glucose Dulbecco’s modified Eagle’s medium (DMEM) (Thermo Fisher Scientific) containing 10% cosmic calf serum (PANC-1 and 8988) (Sigma-Aldrich, St. Louis, MO, USA) or 10% fetal serum (SW1990) (Clark Bioscience, Claymont, DE, USA) and antibiotics (penicillin 100 U/mL, Beyotime; streptomycin 0.1 mg/mL, Beyotime; amphotericin B 2.5 ng/mL, Sangon Biotech, Shanghai, China). Mycoplasma contamination was excluded using the MycoAlert PLUS Mycoplasma detection kit (Lonza Bioscience, Basel, Switzerland) according to manufacturer’s instructions.

### Cell proliferation assay

Cells (1 × 10^5^ cells/well for PaTu-8988t, 2 × 10^5^ cells/well for SW1990 and PANC-1) were seeded in 6-well plates, and subjected to cell counting after 1–4 d on an automated cell counter (Thermo Fisher Scientific).

### Colony formation assay

Cells were seeded in 6-well plates (1 × 10^3^ cells/well), and the medium changed every 3 d. After 14 d, clones were fixed with 4% paraformaldehyde (Shanghai Lingfeng Chemical Reagent Co., Ltd., Shanghai, China) for 30 min, and stained with crystal violet (Beyotime) for an additional 30 min. Cell colonies with a diameter exceeding 0.5 mm were counted using the Image J v 2.4.1.7 (National Institutes of Health, Bethesda, MD, USA).

### Mitochondrial isolation

Mitochondria from cultured cells were isolated as previously described^39^. Cells from 10 individual 100 mm culture dishes at 90% confluence were harvested by trypsinization, pelleted and washed twice with cold PBS. Cells were then homogenized by 30 strokes using a glass dounce tissue grinder (Wheaton, Millville, NJ, USA) and mitochondria were isolated by differential centrifugation^39^.

### Statistical analyses

Data were presented as mean ± standard error of the mean. All experiments were performed in triplicate and at least thrice independently. Statistical significance was evaluated by one-way analysis of variance or independent two-tailed Student’s *t* test using SPSS 21.0 (IBM, Armonk, NY, USA). *P < 0.05, **P < 0.01 and ***P < 0.001.

Detailed experimental procedures can be accessed via Supplementary experimental procedures.

## Supplementary information

Supplementary experimental procedures

Supplementary figure legends

Supplementary table 1

Supplementary figure 1

Supplementary figure 2

Supplementary figure 3

Supplementary figure 4

Supplementary figure 5

Supplementary figure 6

Supplementary figure 7

Supplementary figure 8

Supplementary figure 9

## References

[1] Hanahan D, Weinberg RA (2011). Hallmarks of cancer: the next generation. Cell.

[2] Burk D, Schade AL (1956). On respiratory impairment in cancer cells. Science.

[3] Warburg O (1956). On the origin of cancer cells. Science.

[4] Cho ES, Cha YH, Kim HS, Kim NH, Yook JI (2018). The pentose phosphate pathway as a potential target for cancer therapy. Biomol. Ther..

[5] Bansal A, Simon MC (2018). Glutathione metabolism in cancer progression and treatment resistance. J. Cell Biol..

[6] Chen F (2019). Extracellular vesicle-packaged HIF-1alpha-stabilizing lncRNA from tumour-associated macrophages regulates aerobic glycolysis of breast cancer cells. Nat. Cell Biol..

[7] Ma L (2018). Breast cancer-associated mitochondrial DNA haplogroup promotes neoplastic growth via ROS-mediated AKT activation. Int J. Cancer.

[8] Petros JA (2005). mtDNA mutations increase tumorigenicity in prostate cancer. Proc. Natl Acad. Sci. USA.

[9] Tan AS (2015). Mitochondrial genome acquisition restores respiratory function and tumorigenic potential of cancer cells without mitochondrial DNA. Cell Metab..

[10] Birsoy K (2015). An essential role of the mitochondrial electron transport chain in cell proliferation is to enable aspartate synthesis. Cell.

[11] Vincent A, Herman J, Schulick R, Hruban RH, Goggins M (2011). Pancreatic cancer. Lancet.

[12] Santana-Codina N (2018). Oncogenic KRAS supports pancreatic cancer through regulation of nucleotide synthesis. Nat. Commun..

[13] Kang R (2014). The HMGB1/RAGE inflammatory pathway promotes pancreatic tumor growth by regulating mitochondrial bioenergetics. Oncogene.

[14] Sancho P (2015). MYC/PGC-1alpha balance determines the metabolic phenotype and plasticity of pancreatic cancer stem cells. Cell Metab..

[15] LeBleu VS (2014). PGC-1alpha mediates mitochondrial biogenesis and oxidative phosphorylation in cancer cells to promote metastasis. Nat. Cell Biol..

[16] Zhou C (2018). Oncogenic HSP60 regulates mitochondrial oxidative phosphorylation to support Erk1/2 activation during pancreatic cancer cell growth. Cell Death Dis..

[17] Huttemann M, Jaradat S, Grossman LI (2003). Cytochrome c oxidase of mammals contains a testes-specific isoform of subunit VIb-the counterpart to testes-specific cytochrome c?. Mol. Reprod. Dev..

[18] Barretina J (2012). The cancer cell line encyclopedia enables predictive modelling of anticancer drug sensitivity. Nature.

[19] Gaude E (2018). NADH shuttling couples cytosolic reductive carboxylation of glutamine with glycolysis in cells with mitochondrial dysfunction. Mol. Cell.

[20] Sullivan LB (2015). Supporting aspartate biosynthesis is an essential function of respiration in proliferating cells. Cell.

[21] Chae S (2013). A systems approach for decoding mitochondrial retrograde signaling pathways. Sci. Signal..

[22] Latorre-Pellicer A (2016). Mitochondrial and nuclear DNA matching shapes metabolism and healthy ageing. Nature.

[23] Stewart TA, Yapa KT, Monteith GR (2015). Altered calcium signaling in cancer cells. Biochim. Biophys. Acta.

[24] Ashton TM, McKenna WG, Kunz-Schughart LA, Higgins GS (2018). Oxidative phosphorylation as an emerging target in cancer therapy. Clin. Cancer Res..

[25] Lonardo E (2013). Metformin targets the metabolic achilles heel of human pancreatic cancer stem cells. PLoS ONE.

[26] Bader DA (2019). Mitochondrial pyruvate import is a metabolic vulnerability in androgen receptor-driven prostate cancer. Nat. Metab..

[27] Maxfield KE (2015). Comprehensive functional characterization of cancer-testis antigens defines obligate participation in multiple hallmarks of cancer. Nat. Commun..

[28] LaMarche AE, Abate MI, Chan SH, Trumpower BL (1992). Isolation and characterization of COX12, the nuclear gene for a previously unrecognized subunit of Saccharomyces cerevisiae cytochrome c oxidase. J. Biol. Chem..

[29] Weishaupt A, Kadenbach B (1992). Selective removal of subunit VIb increases the activity of cytochrome c oxidase. Biochemistry.

[30] Tsukihara T (1996). The whole structure of the 13-subunit oxidized cytochrome c oxidase at 2.8 A. Science.

[31] Mansilla N, Racca S, Gras DE, Gonzalez DH, Welchen E (2018). The complexity of mitochondrial complex IV: an update of cytochrome c oxidase biogenesis in plants. Int. J. Mol. Sci..

[32] Vidoni S (2017). MR-1S interacts with PET100 and PET117 in module-based assembly of human cytochrome c oxidase. Cell Rep..

[33] Chattaragada MS (2018). FAM49B, a novel regulator of mitochondrial function and integrity that suppresses tumor metastasis. Oncogene.

[34] Rademaker G (2019). Myoferlin contributes to the metastatic phenotype of pancreatic cancer cells by enhancing their migratory capacity through the control of oxidative phosphorylation. Cancers.

[35] Martinez-Ramirez AS, Diaz-Munoz M, Butanda-Ochoa A, Vazquez-Cuevas FG (2017). Nucleotides and nucleoside signaling in the regulation of the epithelium to mesenchymal transition (EMT). Purinergic Signal..

[36] Schumacher D, Strilic B, Sivaraj KK, Wettschureck N, Offermanns S (2013). Platelet-derived nucleotides promote tumor-cell transendothelial migration and metastasis via P2Y2 receptor. Cancer Cell.

[37] Wheaton WW (2014). Metformin inhibits mitochondrial complex I of cancer cells to reduce tumorigenesis. Elife.

[38] Fernandez-Vizarra E (2010). Isolation of mitochondria for biogenetical studies: an update. Mitochondrion.

[39] Viale A (2014). Oncogene ablation-resistant pancreatic cancer cells depend on mitochondrial function. Nature.

