# Supplementary material for: COX6B2 drives metabolic reprogramming toward oxidative phosphorylation to promote metastasis in pancreatic ductal cancer cells
Source: Oncogenesis. 2020 May 15;9(5):51. doi: 10.1038/s41389-020-0231-2 (PMC7229118; doi:10.1038/s41389-020-0231-2)
Supplement: Supplementary file 1 — Supplementary experimental procedures [file 41389_2020_231_MOESM1_ESM.docx]

**Supplementary experimental procedures**

**Pancreatic ductal adenocarcinoma tissue specimens and immunohistochemistry**

Twenty-seven pairs of 5-μm-thick formalin-fixed paraffin-embedded sections of primary PDAC specimens and paired adjacent normal pancreatic tissues were obtained from patients at People's Hospital of Hangzhou, Zhejiang, China. Similarly, pairs of matched PDAC tumor and adjacent normal tissues were obtained from patients at the First Affiliated Hospital of Wenzhou Medical University (Zhejiang, China), and immediately snap frozen in liquid nitrogen following their surgical removal. Access of formalin-fixed paraffin-embedded sections of primary PDAC specimens and paired adjacent normal pancreatic tissues were approved by the Ethical Committee of the First Affiliated Hospital of Wenzhou Medical University and People's Hospital of Zhejiang. Informed consents were obtained from all subjects. All experimental methods were carried out in accordance with approved guidelines of the Wenzhou Medical University.

Tissue sections were mounted on slides, treated with xylene (Changshu Yangyuan Chemical Co., Ltd, Jiangsu, China), rehydrated in a graded series of alcohol–water solutions and then subjected to heat treatment in 10 mM citric acid (pH 6.0) for 25 min to restore antigens ^1^. Endogenous peroxidase activity was quenched using a 0.3 % H_2_O_2_ solution. Sections were blocked with 10% goat serum (Absin Bioscience Inc, Shanghai, China) for 2 h at 25 °C and incubated with primary anti-COX6B2 (1:50; #PA5-50213, Thermo Fisher Scientific, Waltham, MA, USA) antibody at 4 °C overnight. Sections were stained with diaminobenzidine (DAB, Beyotime, Shanghai, China) to observe the signal, counterstained with hematoxylin, dehydrated in ethanol, clarified in xylene, and then sealed with neutral resin (Sinopharm Chemical Reagent Co., Ltd, Shanghai, China). Protein expression levels were analyzed by calculating the integrated optical density (IOD) for each stained area using Image-Pro Plus 6.0 (Media Cybernetics, Rockville, MD, USA) as previously described ^2^, and mean optimal density (MOD) was calculated as IOD/Area.

**Plasmids, siRNAs, and generation of stable cell lines**

For generating the *COX6B2* knock down (KD), the COX6B2 shRNA sequences were used as follows: shCOX6B2-1, 5′-GCAGCCCTGCGAGTACTATTT-3′ (ORF region); shCOX6B2-2, 5′-GCCTTCGAGTTCCTTGTTTCC-3′ (3′-UTR region). Consecutively, pLKO.1-puro with shRNAs and packing plasmids (pSPAX2 and pMD2G) were co-transfected into HEK 293T cells. Accordingly, SW1990, PANC-1 and 8988 cells were infected with lentivirus-containing medium from the culture of 293T cells. For *COX6B2* re-expression and over-expression, *COX6B2* (NM_001369798.1) MYC-tagged at the C-terminal was synthesized and cloned into the pLV[Exp]-EGFP:T2A:Hygro-EF1A lentiviral vector (Cyagen, Guangdong, Guangzhou, China). Cells with stable expression of *COX6B2* were obtained by infecting cells with the *COX6B2*-containing lentivirus. All cells were cultured in medium containing 4 μg/mL puromycin (Sangon Biotech) or 400 μg/mL hygromycin (Sangon Biotech) for selection of stable cells. A final concentration of 1 μg/mL puromycin or 100 μg/mL hygromycin was used to maintain the respective cultures of stable cells. Control cells were obtained by infecting cells with virus containing empty vectors.

For generation of the *COX6B2* knock-out (KO), 293T cells at 20 % confluence in a 6-well plate were transfected with px330-GFP containing a 20 bp gRNA sequence (5′-CACGCGGAAATAGTACTCGC-3′) using the lipofectamine 3000 Reagent (Thermo Fisher Scientific) according to the manufacturer’s instructions. Single *COX6B2* KO cells were obtained via a strategy of limited dilutions.

Knockdown of *P2X7* was achieved by transient transfection of 8988 cells with stealth RNAi duplex constructs (RiboBio, Guangzhou, Guangdong, China) using the lipofectamine RNAiMAX (Thermo Fisher Scientific) according to the manufacturer’s instructions. The siRNA sequence of the *P2X7* KD was 5′-GCGGAATAATGGGCATTGA-3′. The siRNA sequence for control cells was ordered from RiboBio.

**Xenograft experiment**

All animal experiments were performed in accordance with the Guide for Care and Use of Laboratory Animals outlined in the Animal Ethics Committee of Wenzhou Medical University. Six-week-old nude mice (Beijing Vital River Laboratory Animal Technology Co., Ltd, Shanghai, China) were injected subcutaneously under the right shoulder with 5 × 10^6^ PANC-1 or 8988 cells, 0.2 mL mixture (1:1) of PBS (phosphate buffer saline) and Matrigel (Corning, Corning, NY, USA) each mouse. Tumor volume (mm^3^) defined as (length × width^2^) × 0.5236 was measured using a caliper 3 times a wk until the end of the experiment. After 8 (8988) or 9 wk (PANC-1), all mice were sacrificed to measure the final tumor weight.

***In vivo* metastatic experiment**

4 × 10^6^ PANC-1 cells were injected (tail vin) into six-week-old nude mice (n = 5 mice per group). 11 weeks later, all mice were sacrificed and dissected. The visible metastatic tumor nodules in the lung, liver, gut, mesentery, pancreatic, axillary lymph nodes and pleura of each mouse were counted. All solid organs were harvested and fixed with 4 % paraformaldehyde (24 h), dehydrated in a graded series of alcohol–water and dimethylbenzene solutions. Then, tissues were paraffin embedded, and 5 μm sections were cut and mounted on slides. After that, sections were rehydrated in dimethylbenzene and a graded series of alcohol–water solutions, and counterstained with hematoxylin, dehydrated in ethanol, clarified in xylene, and then sealed with neutral resin. Histopathological images were captured (40×, scale bar 50 μm, 100×, scale bar 20 μm) and analyzed with microscope.

**Wound healing assay**

Cells were seeded in 6-well plates and cultured to form a monolayer of cells at 90 % confluence. Wounds were made using a 10 µL-pipette tip and gently washed with pre-warmed PBS. Wounds were photographed immediately (0 h), as well as at 12 and 24 h. The exposed area at 0 h and 24 h was analyzed using the Image J software (NIH). The wound healing capacity was calculated using the format (exposed area at 0 h - exposed area at 24 h)/exposed area at 0 h. Experiments were performed in triplicate and thrice independently, and at least 5 images/well were captured under a microscope (Nikon, Tokyo, Japan).

**Trans-well assay**

Cell invasion assays were performed using a trans-well chamber (Corning). The inserts were coated with 30 μL Matrigel (1:9 dilution; Corning). Consecutively, 5 × 10^4^ cells/well were resuspended in 200 μL serum-free DMEM and added into the upper chamber of trans-well inserts. Cells tended to penetrate through the Matrigel and translocate toward the lower chamber, where 600 μL DMEM containing 10 % FBS was added. After 22 h of incubation, invading cells were fixed with 4 % paraformaldehyde and stained with crystal violet dye. Invading cells were counted in 3 randomly selected high-power fields using a light microscope (Nikon). Cell migration assays were performed as above, without making use of the Matrigel inserts.

**Immunofluorescence staining**

For the IF staining 2 × 10^4^ cells/well were seeded into 24-well plates containing glass coverslips (WHB, Shanghai, China). After growing on coverslips for about 24 h, cells were fixed with 4 % paraformaldehyde for 10 min at 25 °C, and permeabilized with 100 mΜ digitonin (Sigma-Aldrich) for 10 min. Subsequently, cells were blocked with 10 % goat serum (Absin Bioscience Inc) in PBS for 30 min, and incubated with anti-F-actin antibody (1:100; #ab205, Abcam, Cambridge, UK) at 4 °C overnight. Next, cells were incubated with a fluorescently labeled IgG-Alexa Fluor 488 secondary antibody (1:500; #8878, Cell Signaling Technology, Danvers, MA, USA) for 2 h at 25 °C in the dark, and a drop of antifade mounting medium with DAPI (Beyotime) was used to mount the coverslips. Finally, at least 5 randomly selected fields of each coverslip were photographed using a confocal laser microscope (Nikon). Quantitative analysis of fluorescence intensity was analyzed by calculating the integrated optical density (IOD) of each stained area using the Image J software (NIH).

**Enzyme activity assay**

Mitochondria isolated from cultured cells were used to measure the activity of OXPHOS complexes (complex I-V) according to published protocols ^3^. The activity of complex I (CI) was detected using a buffer containing 12.5 mM potassium phosphate buffer (pH 7.4), 5 mM NaN_3_ (sodium azide; EKER, Shanghai, China), 10 μM DB (decylubiquinone; Sigma-Aldrich), 130 μM NADH (nicotinamide-adenine-dinucleotide; Sigma-Aldrich) and 10 μg/mL antimycin A (Sigma-Aldrich). The activity of CII was detected using a buffer containing 25 mM potassium phosphate buffer (pH 7.4), 8 μg/mL antimycin A, 20 mM succinate and 50 μM DCPIP (2,6-dichlorophenolindophenol; Sigma-Aldrich), 12 μM rotenone (Sigma-Aldrich), and 20 μM DB. The activity of CIII was detected using a buffer containing 12.5 mM potassium phosphate buffer (pH 7.4), 225 μg/mL DDM (n-dodecylβ-D-maltoside), 50 μM of oxidized cytochrome c (Sigma-Aldrich), 200 μM EDTA (Sigma-Aldrich), and 50 μM DBH2 (reduced decylubiquinone; Sigma-Aldrich). The activity of CIV was detected using a buffer containing 10 nM potassium phosphate buffer (pH 7.4), 225 μg/mL DDM, and 6 mg/mL of reduced cytochrome c (Sigma-Aldrich). The activity of CV was detected using a buffer containing 0.6 mM EGTA (ethylenebis (oxyethylenenitrilo) tetraacetic acid; Sigma-Aldrich), 0.03 M Tris-HCl (Tris (hydroxymethyl) aminomethane; Sigma-Aldrich), 0.31 mM NADH, 0.38 mM PEP (phosphoenolpyruvate; Sigma-Aldrich), 3.85 μg/mL antimycin A, 2.65 mM MgCl2 (magnesium chloride; Sigma-Aldrich), 0.15 μg/mL LDH (lactate dehydrogenase; Sigma-Aldrich), 85 μg/mL PK (pyruvate kinase; Sigma-Aldrich), and 2.5 mM ATP (adenosine triphosphate; Sigma-Aldrich). Kinetic curves for the activities of OXPHOS complexes were recorded using a Varioskan™ Flash Multimode Reader (Thermo Fisher Scientific) at 340, 600, 550, 550, and 340 nm for 5 min at 37 °C, respectively. The activity of citrate synthase was used as internal control and was detected at 412 nm using a buffer containing 12.5 mM potassium phosphate buffer (pH 7.4), 225 μg/mL DDM, 1 mM DTNB (5,5'-dithiobis-2-nitrobenzoic acid; Sigma-Aldrich), 68 μM acetyl-coenzyme A (Sigma-Aldrich), and 0.5 mM OAA (oxaloacetate; Sigma-Aldrich).

**Blue native polyacrylamide gel electrophoresis, sodium dodecyl sulfate gel electrophoresis, and immunoblotting**

Total proteins were extracted from cells using the RIPA lysis buffer (Cell Signaling Technology). Mitochondrial membrane proteins were isolated from whole cells with 2 % DDM (Sigma-Aldrich) or 2 % digitonin (Merck KGaA, Darmstadt, Germany). Proteins separated by blue native polyacrylamide gel electrophoresis (BNG) or sodium dodecyl sulfate (SDS)-PAGE were transferred to 0.22 μm polyvinylidene difluoride (PVDF) membranes (Bio-Rad, Hercules, CA, USA). Proteins were probed using anti-Grim19 (1:1000; #ab110240, Abcam), anti-SDHA (1:2000; #ab14715, Abcam), anti-UQCRC2 (1:1000; #ab14745, Abcam), anti-MT-COI (1:1000; #ab14705, Abcam), anti-ATP5A (1:2000; #ab14748, Abcam), anti-VDAC (1:1000; #4661, Cell Signaling Technology), anti-TOM70 (1:1000; #4527-1, Proteintech, Wuhan, China), anti-COX6B2 (1:1000; #ab134960, Abcam), anti-MYC (1:1000; #2272, Cell Signaling Technology), anti-βactin (1:2000; # sc-47778, Santa Cruz Biotechnology, Delaware, USA) and anti-GAPDH (1:2000; sc-47724, Santa Cruz Biotechnology) antibodies, and then incubated with a horseradish peroxidase-conjugated anti-rabbit (1:2000; #7074, Cell Signaling Technology) or anti-mouse IgG secondary antibody (1:2000; #7076, Cell Signaling Technology). Signals were detected using the Super Signal West Pico chemiluminescent substrate (Thermo Fisher Scientific). Integrated optical density quantification was performed using the Gel-Pro Analyzer 4.0 software (Media Cybernetics, Rockville, MD, USA).

**Reagents**

N-Acetyl-L-cysteine (NAC), Brilliant Blue-G (BBG), chloramphenicol (CAP), Compound C, ATP and glucose were purchased from Sigma-Aldrich. NaN_3_, metformin, apyrase, and RB2 were purchased from Shanghai EKEAR Biotechnology (Shanghai, China), Sangon Biotech, New England Biolabs (Ipswich, MA, USA), and MedChemExpress (Monmouth Junction, NJ, USA), respectively.

**Measurement of oxygen consumption**

The oxygen consumption rate (OCR) of PDAC cells was analyzed using the Seahorse Bioscience XF24 Extracellular Flux Analyzer (Seahorse Bioscience, Santa Clara, CA, USA). Briefly, 3 × 10^4^ cells/well with 250 μL medium were seeded in seahorse 24-well plates and cultured overnight. Before measurement, cells were gently washed with assay medium, cultured at 37 °C in a CO_2_-free incubator with 675 μL assay medium per well for 1 h. After calibration of the analyzer, the basal respiration, ATP production (with 1 μΜ oligomycin; BBI Life Science, Shanghai, China), maximal respiration (with 0.5 μΜ carbonyl cyanide-p-trifluoromethoxyphenylhydrazone (FCCP); Sigma) and spare capacity (with 0.5 μΜ antimycin A and 0.5 μΜ rotenone; Sigma) were measured.

**Measurement of lipid droplet**

For this assay, 2 × 10^4^ cells/well were seeded in 24-well plates containing glass coverslips. After 24 h, cells were stained with 2 μM BODIPY 581/591 C11 (Thermo Fisher Scientific) in serum-free DMEM for 30 min. Coverslips were mounted with a drop of antifade mounting medium containing DAPI (Beyotime), and at least 5 randomly selected fields of each coverslip were photographed using a confocal laser microscope (Nikon). Quantitative analysis of the fluorescence intensity was analyzed by calculating the IOD of each stained area using the Image J software (NIH).

**ATP measurement**

ATP content were determined using the ATP measurement kit (Molecular Probes, Carlsbad, CA, USA) according to the manufacturer’s instruction. Approximately 1 × 10^6^ cells were harvested and pelleted by trypsinization and washed twice with cold PBS. Cells were then resuspended and boiled in 100 μL boiling buffer (100 mM Tris (Sigma-Aldrich), 4 mM EDTA (Sigma-Aldrich), adjusted to pH 7.75 with acetic acid) for 90 s. Supernatants were retrieved by centrifugation at 10, 000 × g for 1 min. ATP contents were determined by measuring the luminescence of supernatants mixed with luciferase assay buffer using a Varioskan™ Flash Multimode Reader (Thermo Fisher Scientific).

To measure mitochondrial ATP (Mito-ATP), cells were incubated with 5 mM 2-deoxyglucose (2-DG; Sigma) and 5 mM pyruvate (Sigma) for 2 h prior to measurement. Levels of ATP were normalized with protein concentration and results are presented as the mean of luminescence intensity relative to control cells.

**Measurement of mitochondrial membrane potential**

Mitochondrial membrane potential (MMP) was measured using tetramethylrhodamine (TMRM; Thermo Fisher Scientific). Briefly, cells were cultured in 6-well plates for 2 d and then treated with 30 nM TMRM for 15 min at 37 °C. Cells were subsequently washed 3 times with PBS and observed using a fluorescence microscope (Nikon). Fluorescence images were captured randomly (10 images/well). The TMRM fluorescence intensity was analyzed by calculating the IOD of each stained area using the Image J software (NIH).

**Reactive oxygen species measurement**

Mitochondrial ROS were measured according to a previously published protocol ^4^. In short, cells were washed with Hank’s buffered salt solution (HBSS) and incubated with DMEM containing 5 μM MitoSOX (Thermo Fisher Scientific) for 20 min at 37 °C. Cells were washed with HBSS twice and fluorescence was record using a Varioskan™ Flash Multimode Reader (Thermo Fisher Scientific) with excitation at 488 nm and emission at 530 nm. Intracellular ROS were determined by staining cells with 20 μM 6-chloromethyl-2',7'-dichlorodihydrofluorescein diacetate, acetyl ester (carboxy-H2DCFDA; Thermo Fisher Scientific) with excitation at 535 nm and emission at 575 nm. Data were normalized with protein concentration and results are presented as the mean of fluorescence intensity relative to control cells.

**Mitochondrial calcium measurement**

After growing in 6-well plates for about 24 h, cells (approximately 1 × 10^6^ cells/well) were stained with 5 μM Rhod-2 AM (Abcam) in serum-free DMEM for 30 min at 37 °C and then washed 3 times and re-suspended with PBS. Fluorescence intensity was measured using a Varioskan™ Flash Multimode Reader (Thermo Fisher Scientific) with excitation at 552 nm and emission at 581 nm. Data were normalized with protein concentration and results are presented as the mean of fluorescence intensity relative to control cells.

**Quantitative real-time PCR**

Total RNA was extracted from cells as previously described ^5^ and reverse-transcribed into cDNA using an RNA reverse transcription kit (Takara Biotechnology, Dalian, China). Quantitative real-time PCR was performed using primers targeted to the mRNA of *β-actin*, *COX6B2*, and *P2X7* using the SYBR Green qPCR Master Mix (Takara Biotechnology) on a StepOne Real-Time PCR System (Thermo Fisher Scientific). Primers used in the analysis were as follows:

β-actin: 5′-GACCTGTACGCCAACACAGT-3′ (forward)

5′-AGTACTTGCGCTCAGGAGGA-3′ (reverse)

COX6B2: 5′-CAGCCAGAACCAGATCCGTA-3′ (forward)

5′-CGAGTGGTACACGCGGAAAT-3′ (reverse)

P2X7: 5′-AAGCTGTACCAGCGGAAAGAG-3′ (forward)

5′-CTGAATTCCTTTGCTCTGCGG-3′ (reverse)

Data were analyzed using the 2(-ΔΔCT) method as previously described ^4^.

**Public data acquisition and analysis**

The gene expression data (TOIL RSEM expected count) processed by the Toil pipeline based on raw GTEx and TCGA RNA-sequencing data to correct for batch effects and to allow for the merging of samples across GTEx and TCGA datasets for pan-analyses was downloaded from the UCSC Xena browser website (https://xenabrowser.net/). Custom Perl script was used to extract the pancreatic ductal adenocarcinoma (PDAC) data for subsequent analysis. A total of 348 samples (containing 178 cancer and 170 normal samples) were extracted, and genes with high missing values (more than 20 % of samples) were filtered out. Differential expression analysis was performed using the DESeq2 R package ^6^. DESeq2 provided statistical routines for determining the differential expression in digital gene-expression data using a model based on a negative binomial distribution. Resulting *P* values were adjusted using the Benjamini-Hochberg approach to control for false-discovery rates ^7^. Genes found using DESeq2 that featured an adjusted *P* < 0.05 were regarded as differentially expressed genes (DEGs). Heatmaps were drawn using the R package heatmap.

**Cell preparation and transcriptome profiling**

Total RNA was isolated from *COX6B2* KD 8988 cells and control cells (n = 3) using an RNeasy Mini Extraction kit (Qiagen, [Valencia](http://www.yelp.com/biz/qiagen-valencia), CA, USA), and the mRNA from 20 μg of total RNA was purified using poly-T-attached magnetic beads. After fragmenting, the mRNA first-strand cDNA was synthesized and then sequenced using an Illumina HiSeq 2000 platform (Illumina, San Diego, CA, USA), as previously described ^8^.To obtain clean reads, reads containing adaptors, reads containing adaptor sequences and poly-N, and low-quality reads were removed from the raw data. Reference-genome and gene-model annotation files were downloaded directly from genome website (ftp://ftp.ensembl.org/pub/release-75). The paired-end clean reads were aligned to the built reference genome, using STAR (v2.5.1b). HTSeq v0.6.0 was used to count the reads mapped to each gene. The DESeq2 R package was used to normalize the gene count. Gene Set Enrichment Analysis (GSEA) was done using the GSEA (v4.0.1) software ^9^ distributed from the MIT Broad institute website (http://software.broadinstitute.org/gsea), and the false discovery rate (FDR) threshold was speciﬁed at 0.25.

**Cell preparation and metabolomic proﬁling**

*COX6B2* KD 8988 cells and control cells (n = 6) were plated in regular medium. After 48 h, approximately 1 × 10^7^ cells were collected and snap frozen in liquid nitrogen. Samples were sent to Metabolon, Inc. (Durham, NC, USA) for metabolomic profiling using an untargeted, gas chromatography-mass spectrometry and liquid chromatography-mass spectrometry (GC-MS and LC-MS)-based metabolomic quantification protocol ^10^. Principal component analysis (PCA) using R software was used to display the overall differences. For the multivariate model analysis, between-group differences in the abundance of metabolites identified by mass spectrometry (liquid chromatography–mass spectrometry and gas chromatography–mass spectrometry) were assessed by Welch’s two-sample *t-test*. Differences were considered statistically significant at *P* < 0.05.

**References**

[1] J. Zhu. *et al*, NF-kappaB p65 Overexpression Promotes Bladder Cancer Cell Migration via FBW7-Mediated Degradation of RhoGDIalpha Protein, Neoplasia, 19 (2017) 672-683.

[2] C.J. Wang. *et al*, Survivin expression quantified by Image Pro-Plus compared with visual assessment, Appl Immunohistochem Mol Morphol, 17 (2009) 530-535.

[3] M.A. Birch-Machin, D.M. Turnbull, Assaying mitochondrial respiratory complex activity in mitochondria isolated from human cells and tissues, Methods Cell Biol, 65 (2001) 97-117.

[4] H. Fang. *et al*, Mitochondrial DNA haplogroups modify the risk of osteoarthritis by altering mitochondrial function and intracellular mitochondrial signals, Biochim Biophys Acta, 1862 (2016) 829-836.

[5] H. Fang. *et al*, mtDNA Haplogroup N9a Increases the Risk of Type 2 Diabetes by Altering Mitochondrial Function and Intracellular Mitochondrial Signals, Diabetes, 67 (2018) 1441-1453.

[6] M.I. Love, W. Huber, S. Anders, Moderated estimation of fold change and dispersion for RNA-seq data with DESeq2, Genome Biol, 15 (2014) 550.

[7] S. Chae. *et al*, A systems approach for decoding mitochondrial retrograde signaling pathways, Science signaling, 6 (2013) rs4.

[8] C. Zhou. *et al*, Oncogenic HSP60 regulates mitochondrial oxidative phosphorylation to support Erk1/2 activation during pancreatic cancer cell growth, Cell Death Dis, 9 (2018) 161.

[9] A. Subramanian. *et al*, Gene set enrichment analysis: a knowledge-based approach for interpreting genome-wide expression profiles, Proc Natl Acad Sci U S A, 102 (2005) 15545-15550.

[10] D.P. Labbe. *et al*, High-fat diet fuels prostate cancer progression by rewiring the metabolome and amplifying the MYC program, Nature communications, 10 (2019) 4358.
