# Supplementary material for: COX6B2 drives metabolic reprogramming toward oxidative phosphorylation to promote metastasis in pancreatic ductal cancer cells
Source: Oncogenesis. 2020 May 15;9(5):51. doi: 10.1038/s41389-020-0231-2 (PMC7229118; doi:10.1038/s41389-020-0231-2)
Supplement: Supplementary file 2 — Supplementary figure legends [file 41389_2020_231_MOESM2_ESM.docx]

**Supplementary figure legends**

**Fig. S1 Profiling of *COX6B2* from public databases**

**(A)** Heatmap of RNA-Seq expression z-scores computed for nuclear encoded OXPHOS genes between pancreatic ductal adenocarcinoma and normal pancreatic tissues from the TCGA and GTEx datasets, respectively.

**(B)** Analysis of *COX6B2* mRNA levels among 30 cancer types in the TCGA database.

**(C)** Analysis of *COX6B2* mRNA levels among 64 cancer cell lines in the CCLE database.

**Fig. S2 Western blot analysis of transfection efficiency**

**(A-C)** Levels of COX6B2 in 3 stable cell lines with knockdown of *COX6B2* (SW1990 (A), PANC-1 (B), and PaTu-8988t (C)).

**(D-E)** Levels of COX6B2 (D) and MYC (E) in *COX6B2* re-expression cells.

**Fig. S3 Metastatic potential of PDAC cells with over-expression of *COX6B2***

**(A and B)** Over-expression of MYC tagged COX6B2 in PANC-1 (A) and 8988 (B) cells.

**(C and D)** Wound healing assays of PANC-1 (C) and 8988 (D) cells compared with control cells.

All data are presented as mean ± SEM (n ≥ 3). **P* < 0.05, ****P* < 0.001.

**Fig. S4 The assembly of complex IV in stable HEK 293T cells with knockout of *COX6B2*.**

**(A)** Western blot analysis of COX6B2 in stable HEK 293T cells with knockout (KO) of *COX6B2* compared with control (Ctrl) cells.

**(B)** 2 % DDM-permeabilized cells analyzed by BNG show mitochondrial monomer complex IV levels in HEK 293T cells with or without knockout of *COX6B2*.

**(C)** 2 % DDM-permeabilized mitochondria analyzed by BNG show sub-complexes IV levels in HEK 293T cells with or without knockout of *COX6B2*. Arrows indicate MT-COI in mature COX (CIV), subassembly intermediates (Sub-CIV) and COX dimers (CIV_2_).

**(D)** 2 % digitonin-permeabilized cells analyzed by BNG show all complex IV levels in HEK 293T cells with knockout of *COX6B2*. Arrows indicate supercomplexes including I+III+IV, III+IV, and dimerized forms of complex IV.

Control cells were infected with corresponding lentiviruses containing empty vectors.

Blots were probed with anti-Grim19 (CI), anti-SDHA (CII), anti-UQCRC2 (CIII), anti-MT-COI (CIV), anti-ATP5A (CV), anti-COX6B2, anti-β-actin, anti-TOM70 and anti-VDAC.

**Fig. S5 Cellular ATP production in PDAC cells with over-expression of *COX6B2***

**(A and B)** Cellular ATP production in PANC-1 (A) and 8988 (B) cells compared with control cells. All data are presented as mean ± SEM (n ≥ 3). **P* < 0.05.

**Fig. S6 Transcriptional analysis of mitochondrial biogenesis markers in PDAC cells with *COX6B2* knockdown**

**(A)** Expression of 13 mtDNA encoded transcriptional products in control 8988 cell and 8988 cells with KD of *COX6B2*.

**(B)** Expression of *RXRA*, *NRF1*, *PGC1α*, and *NRF2* in control 8988 cells and 8988 cells with KD of *COX6B2*.

**Fig. S7 Extracellular acidification rate in PDAC cells with *COX6B2* knockdown**

**Extracellular acidification rate (**ECAR) of control 8988 cells and 8988 cells with *COX6B2* knockdown. Value of ECAR was normalized with cell number.

**Fig. S8 Metabolic features change in PDAC cells with *COX6B2* knockdown**

**(A)** Principal component analysis (PCA) plot for *COX6B2* knockdown (KD) PaTu-8988t cells and control (Ctrl) cells. (n = 6 per cell type).

**(B-F)** Heatmaps of the metabolites involved in glycolysis or gluconeogenesis, (B) purine metabolism, (C) pyrimidine metabolism, (D) amino acid metabolism and (E) the TCA cycle (F) pathway. Metabolites marked in blue, red and black represent downregulated, upregulated and unchanged, respectively, compared with control cells.

**Fig. S9 *COX6B2* promotes PDAC cells metastasis via the ATP/purinergic receptor pathway**

**(A)** Intracellular ROS levels of 3 stable cell lines with knockdown of *COX6B2* compared with corresponding control cells. ROS levels were determined by staining cells with 20 μM carboxy-H2DCFDA diacetate.

**(B)** Mitochondrial ROS levels in the 3 studied cell lines. Cells were stained with 5 μM MitoSOX.

**(C)** AMP levels in *COX6B2* KD PaTu-8988t cells compared with control cells. Data of AMP levels were obtained from the metabolomic proﬁling.

**(D)** GSH/GSSG ratio in *COX6B2* KD PaTu-8988t cells compared with control cells. Data of GSH and GSSG levels were obtained from the metabolomic proﬁling.

**(E)** Mitochondrial calcium levels of *COX6B2* KD PaTu-8988t cells. Fluorescence was measured using a Varioskan™ Flash Multimode Reader and data were normalized to protein concentration.

**(F)** Wound healing assays of *COX6B2* KD PaTu-8988t cells. N-acetyl-L-cysteine (NAC, 5 mM) was used to eliminate ROS.

**(G**) Wound healing assays of *COX6B2* KD PaTu-8988t cells.Compound C (8 μM) was used to inhibit the AMP-activated protein kinase (AMPK) pathways.

All quantiﬁed data are presented as mean ± SEM (n ≥ 3). **P* < 0.05, ***P* < 0.01, and ****P* < 0.001.
