# Supplementary figures and images for: COX6B2 drives metabolic reprogramming toward oxidative phosphorylation to promote metastasis in pancreatic ductal cancer cells
Source: Oncogenesis. 2020 May 15;9(5):51. doi: 10.1038/s41389-020-0231-2 (PMC7229118; doi:10.1038/s41389-020-0231-2)

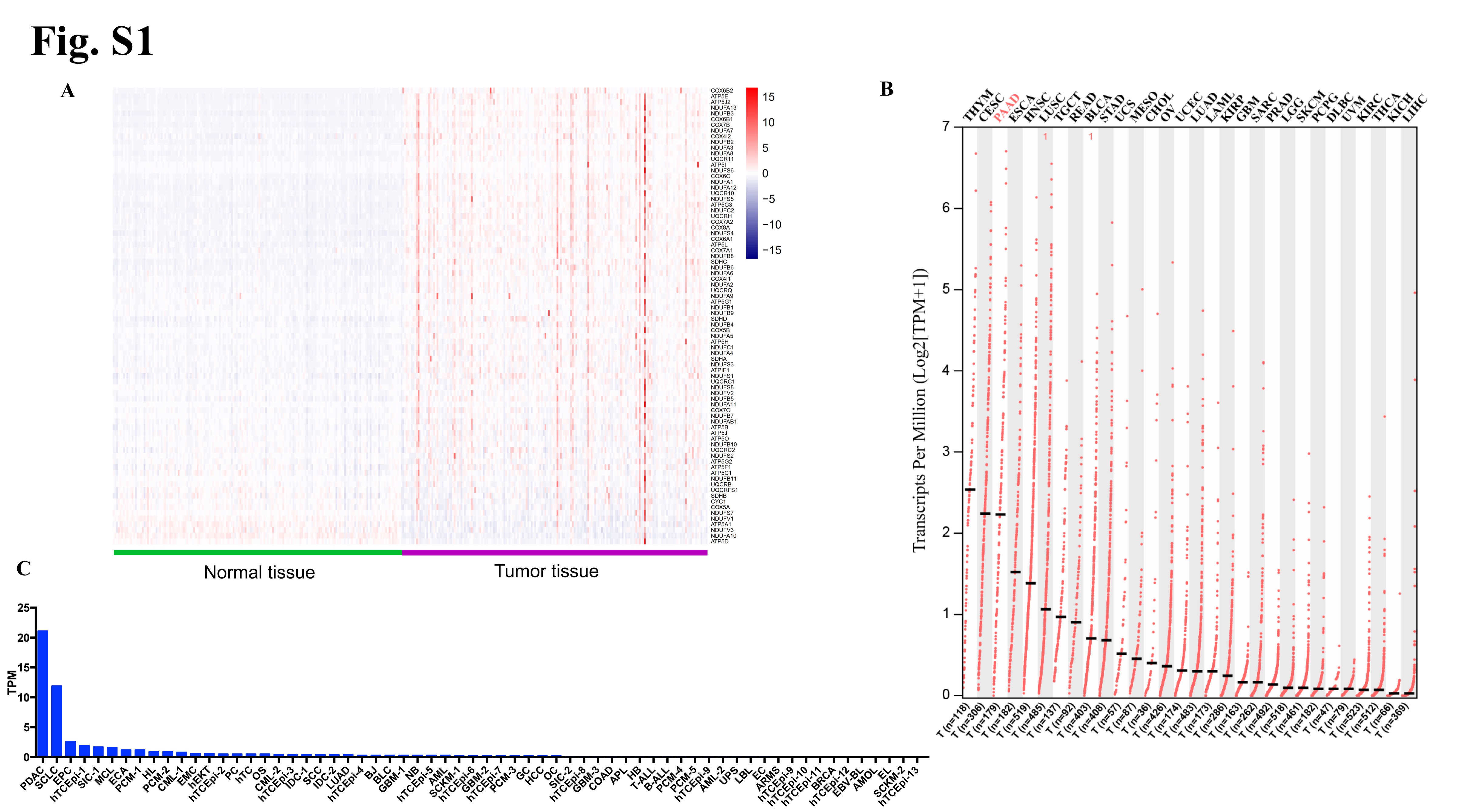

Supplement: Supplementary file 4 — Supplementary figure 1 [file 41389_2020_231_MOESM4_ESM.jpg]

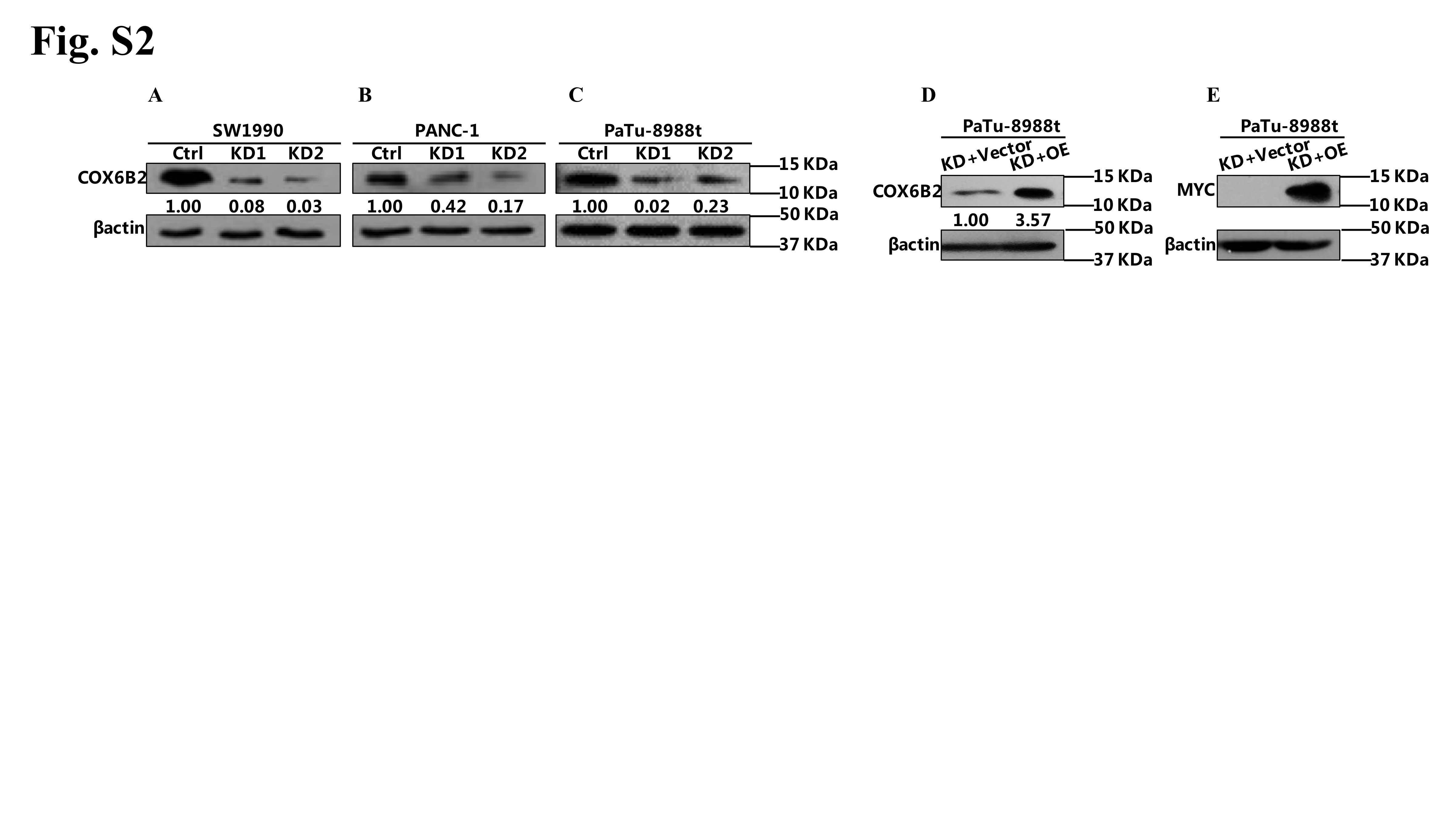

Supplement: Supplementary file 5 — Supplementary figure 2 [file 41389_2020_231_MOESM5_ESM.jpg]

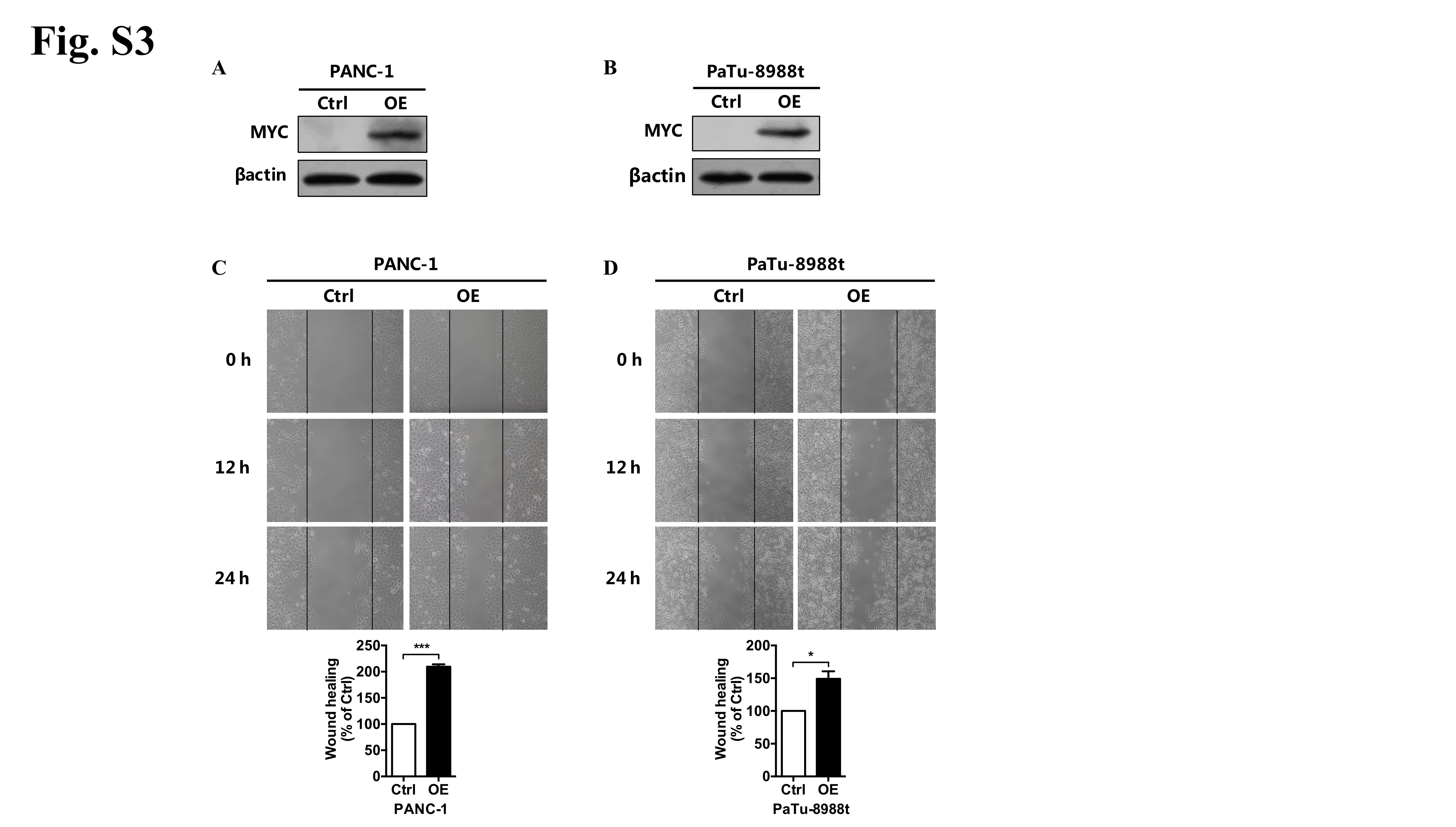

Supplement: Supplementary file 6 — Supplementary figure 3 [file 41389_2020_231_MOESM6_ESM.jpg]

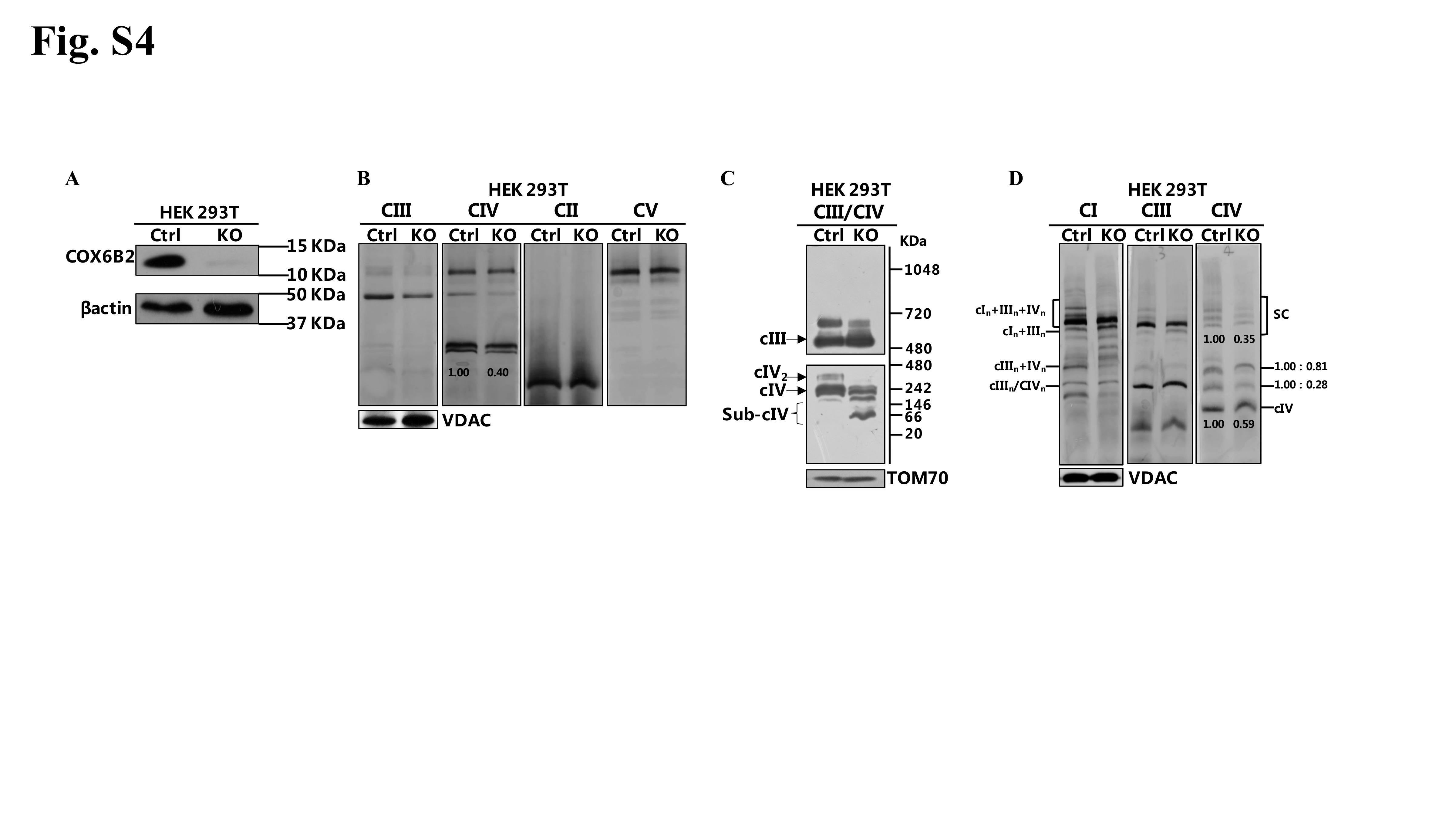

Supplement: Supplementary file 7 — Supplementary figure 4 [file 41389_2020_231_MOESM7_ESM.jpg]

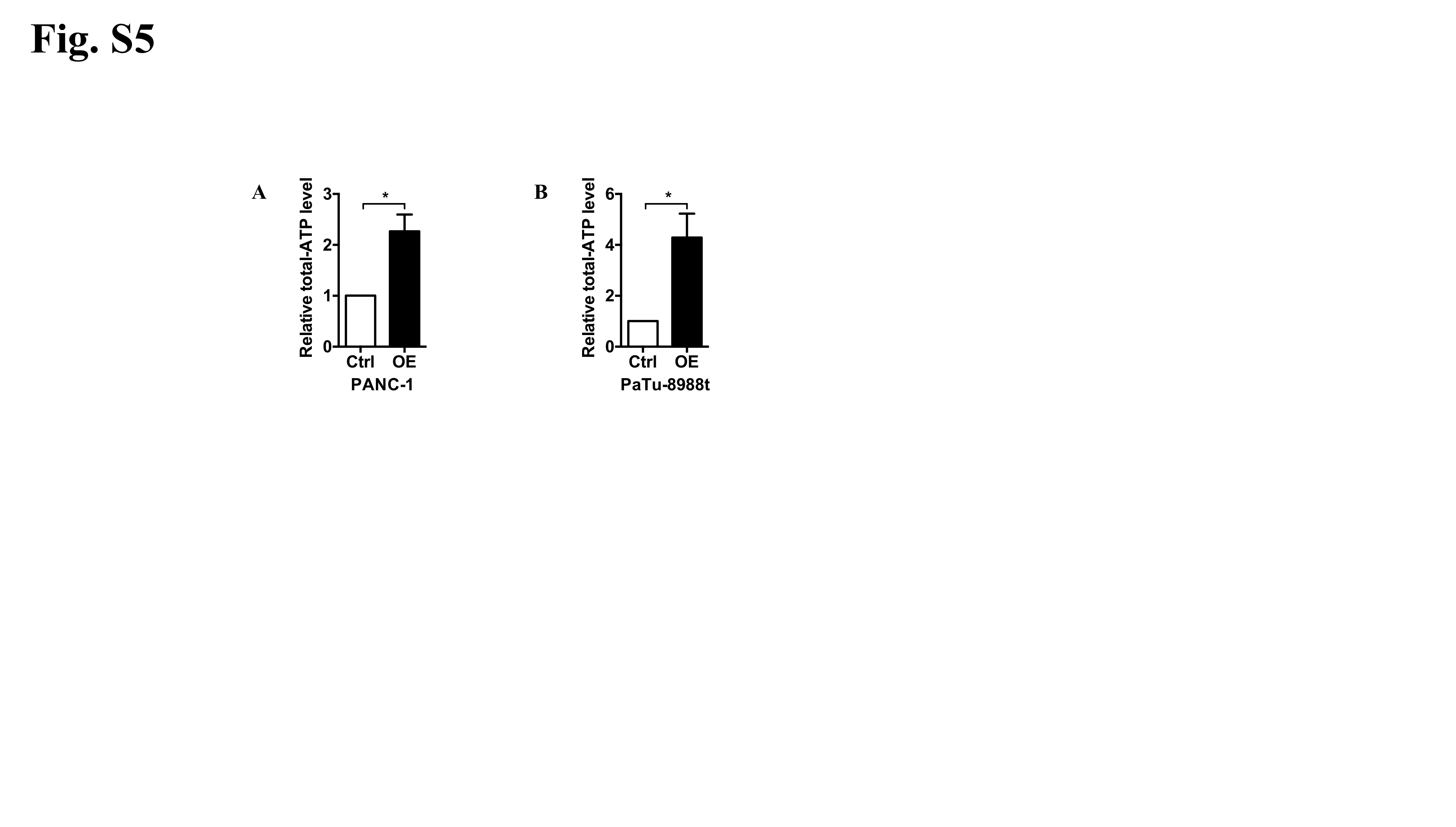

Supplement: Supplementary file 8 — Supplementary figure 5 [file 41389_2020_231_MOESM8_ESM.jpg]

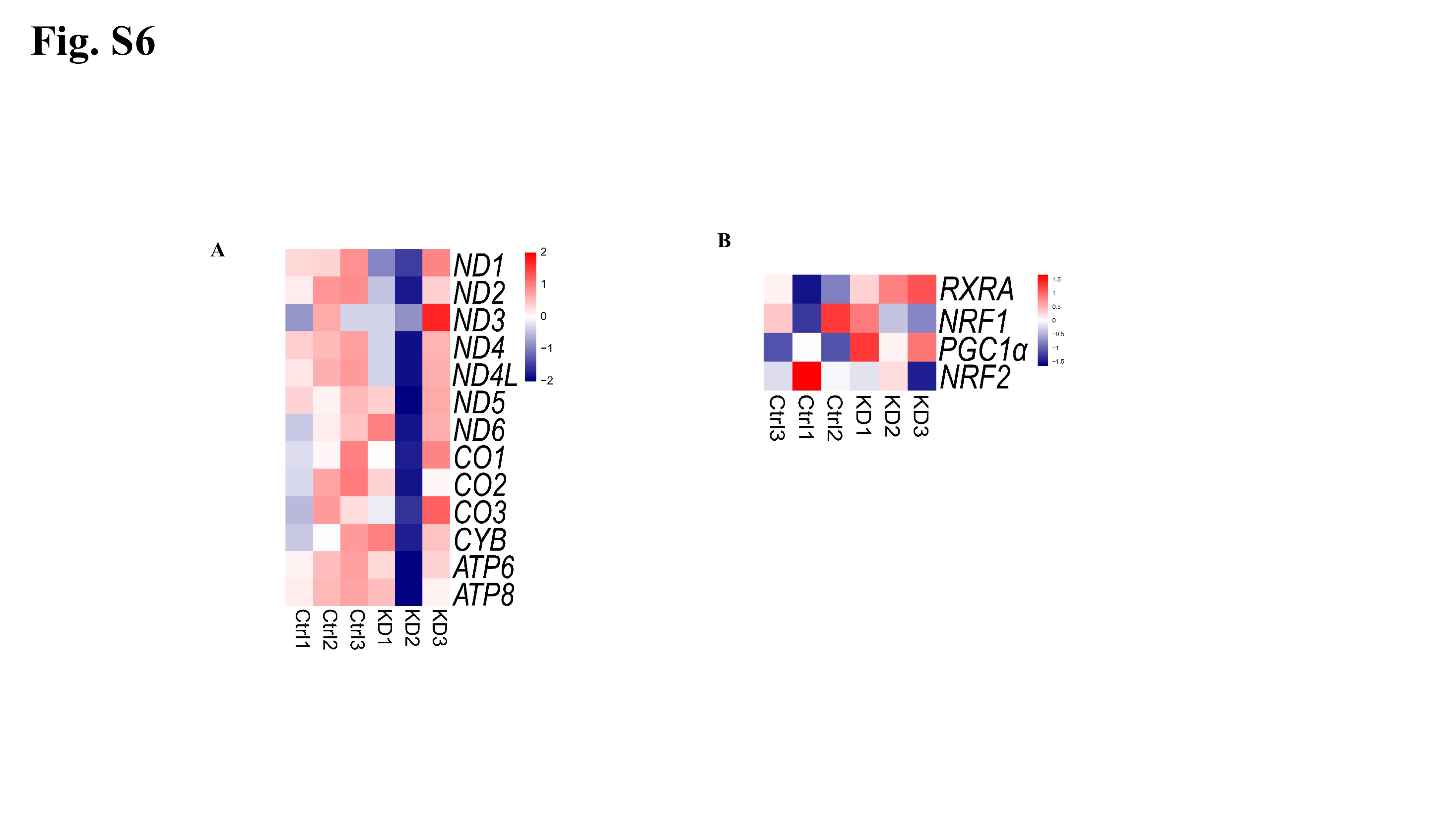

Supplement: Supplementary file 9 — Supplementary figure 6 [file 41389_2020_231_MOESM9_ESM.jpg]

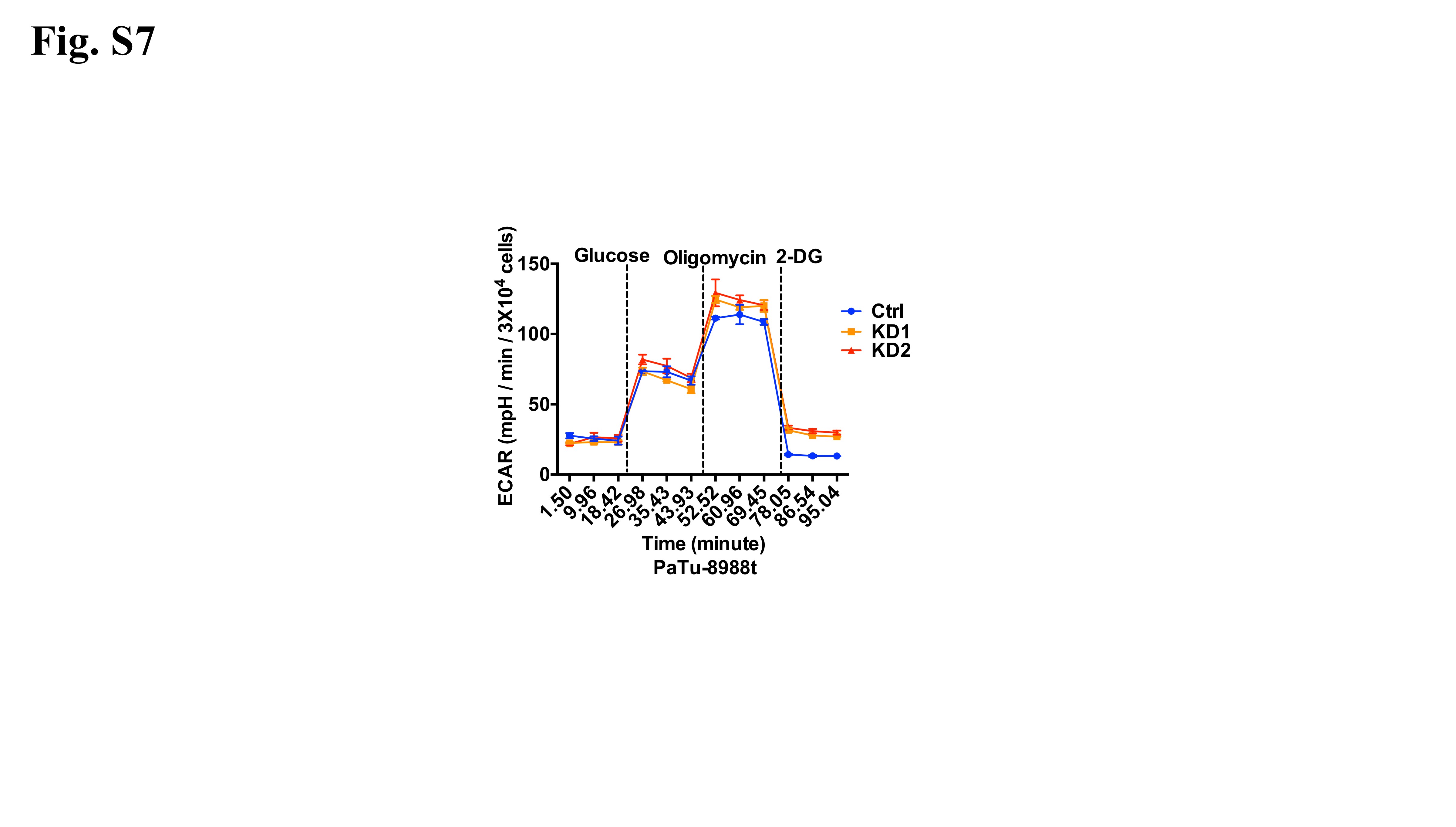

Supplement: Supplementary file 10 — Supplementary figure 7 [file 41389_2020_231_MOESM10_ESM.jpg]

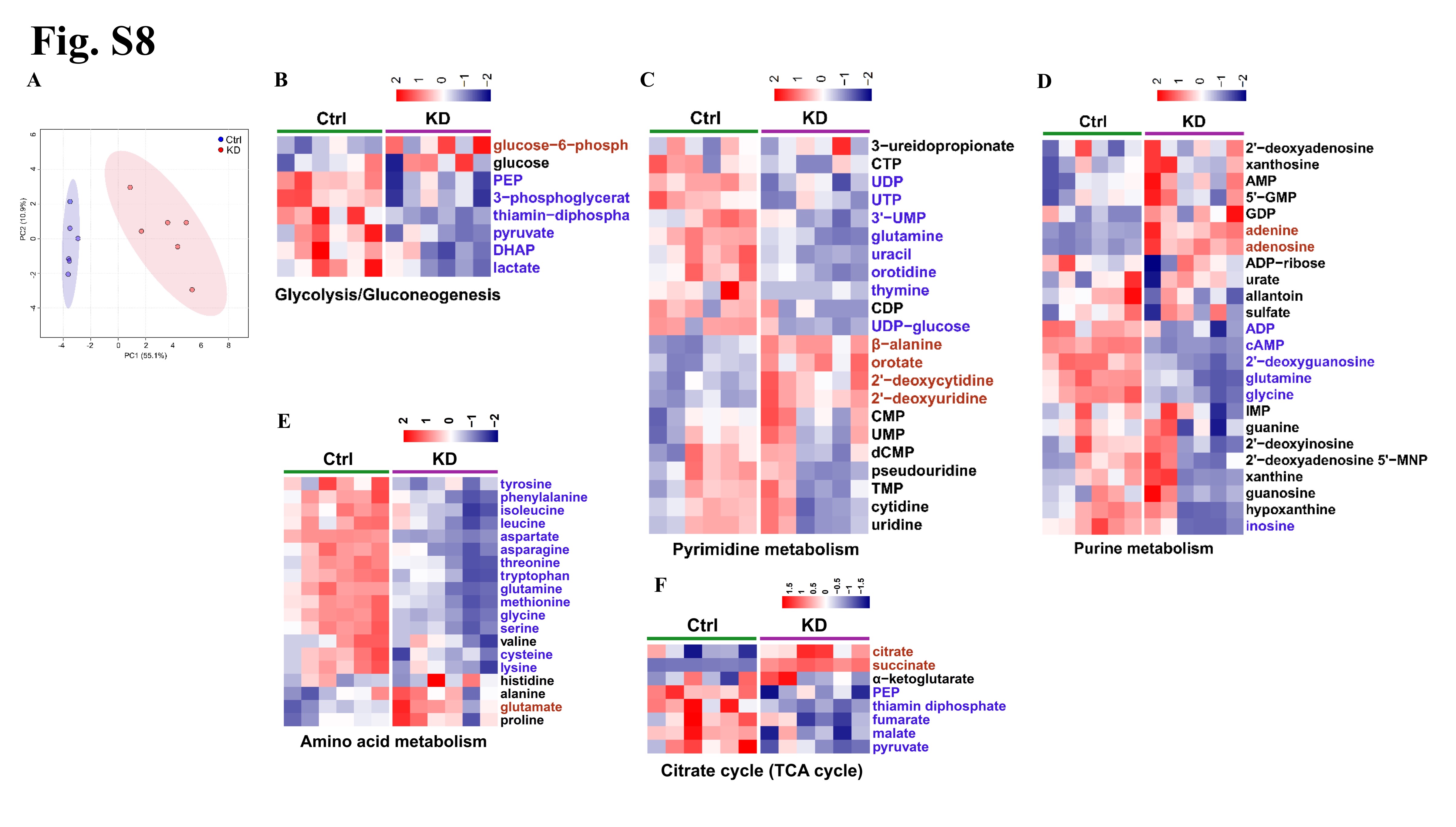

Supplement: Supplementary file 11 — Supplementary figure 8 [file 41389_2020_231_MOESM11_ESM.jpg]

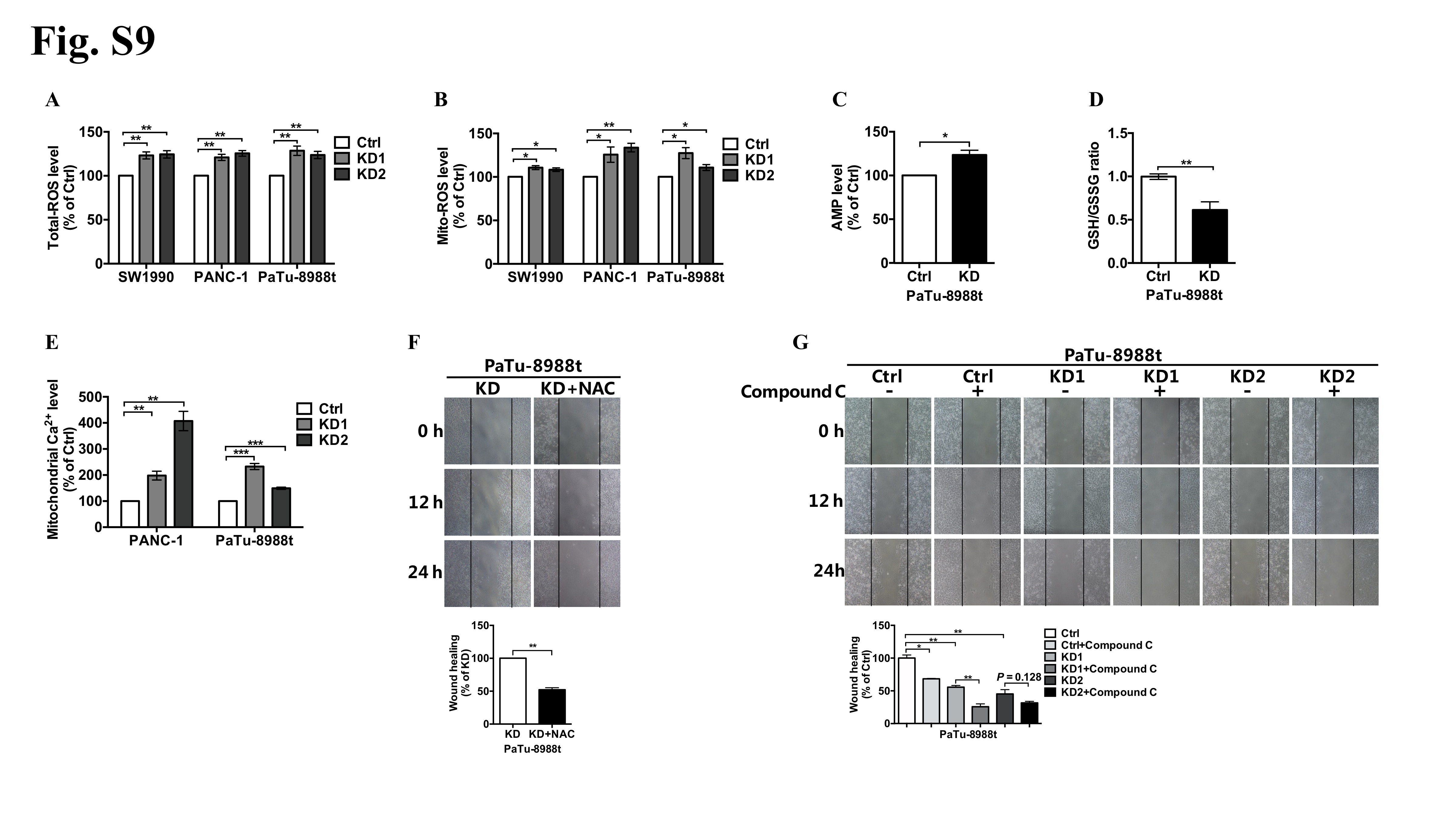

Supplement: Supplementary file 12 — Supplementary figure 9 [file 41389_2020_231_MOESM12_ESM.jpg]
